# Supplementary material for: Tailoring coordination environments of single-atom electrocatalysts for hydrogen evolution by topological heteroatom transfer
Source: Nat Commun. 2024 Mar 30;15:2774. doi: 10.1038/s41467-024-47061-6 (PMC10981667; doi:10.1038/s41467-024-47061-6)
Supplement: Supplementary file 1 — Supplementary Information [file 41467_2024_47061_MOESM1_ESM.pdf]

Supplementary Information

Tailoring coordination environments of single-atom electrocatalysts for hydrogen evolution by topological heteroatom transfer

Table of Contents

Supplementary Figures 1-54
Supplementary Tables 1-8
Supplementary References

Supplementary Figures

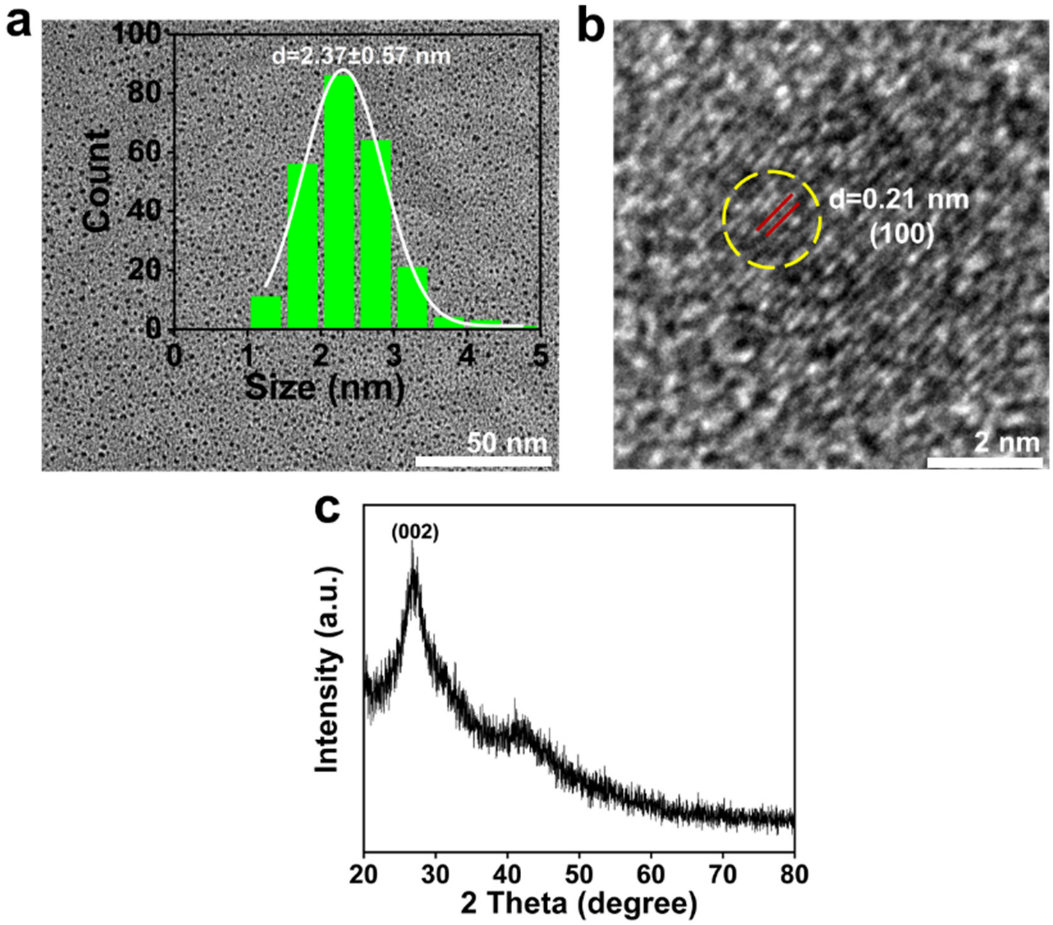

Supplementary Fig. 1: Characterization of NGQDs. (**a**) TEM image with size distribution (inset), (**b**) HRTEM image, and (**c**) XRD pattern of the NGQDs. Transmission electron microscopy (TEM) analysis revealed that the resulting amino and hydroxyl co-functionalized GQDs (NGQDs) exhibited excellent dispersion and a uniform lateral size of 2.3 ± 0.6 nm. The inter-planar distance of 0.35 nm observed in the TEM images corresponded to the (002) plane[1], indicating consistent structural properties.

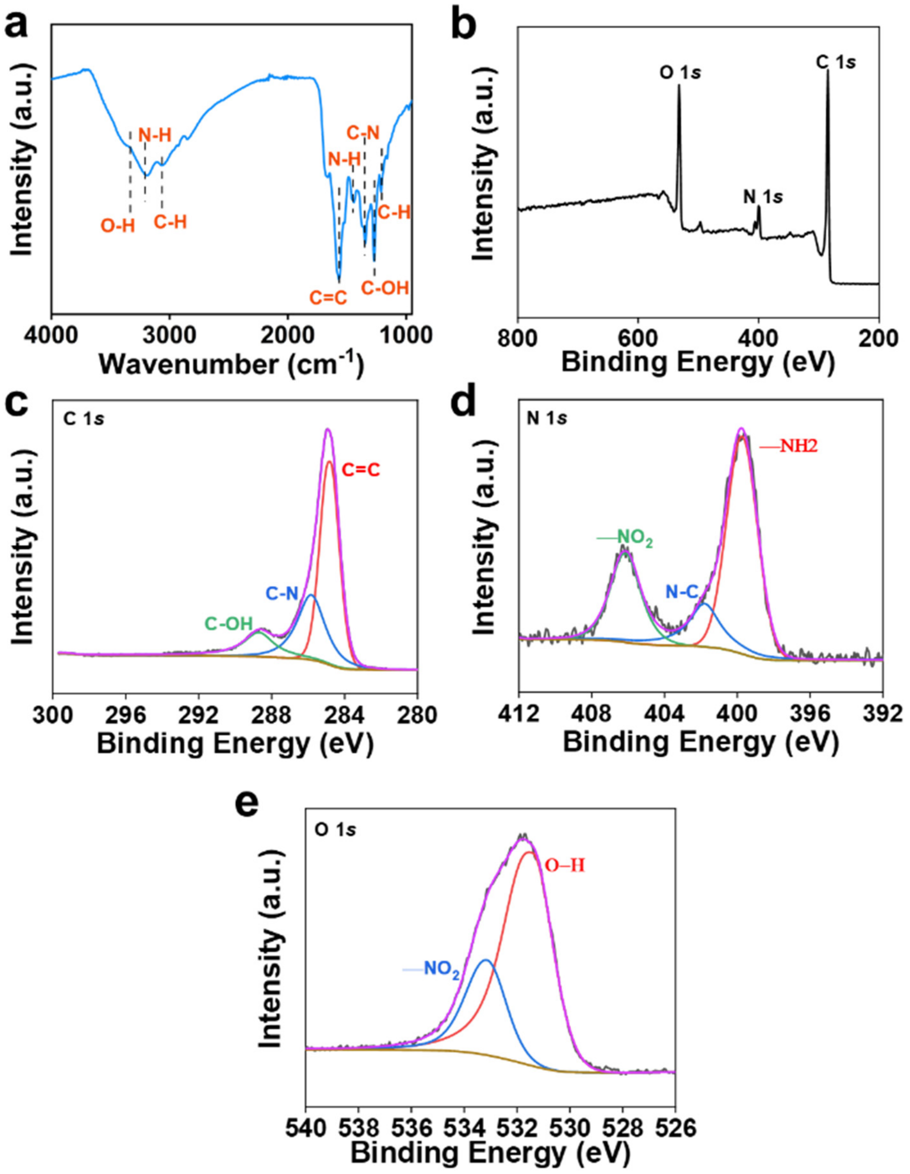

Supplementary Fig. 2: Spectroscopy analysis of NGQDs. (**a**) FT-IR spectrum, (**b**) XPS survey spectrum, (**c**) C 1*s*, (**d**) N 1*s*, and (**e**) O 1*s* spectra of NGQDs.

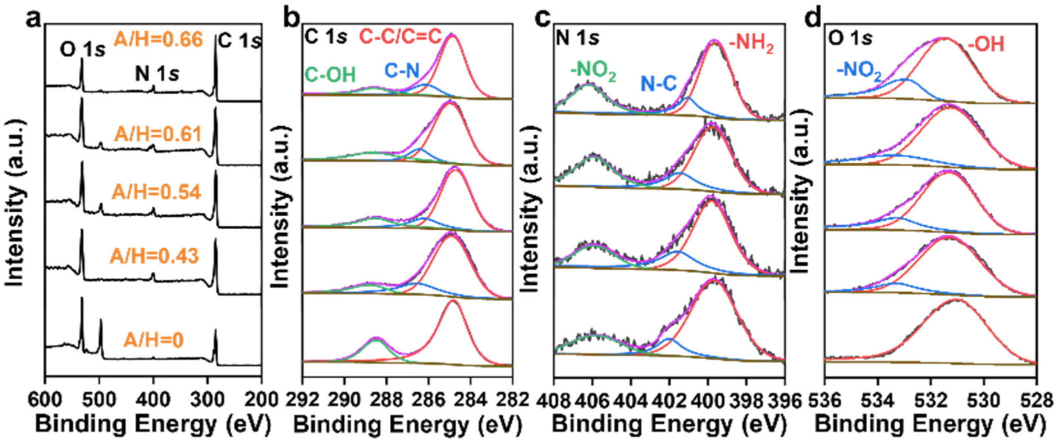

Supplementary Fig. 3: XPS analysis of NGQDs with different A/H. (a) Survey spectra, (b) C 1*s*, (c) N 1*s*, and (d) O 1*s* spectra.

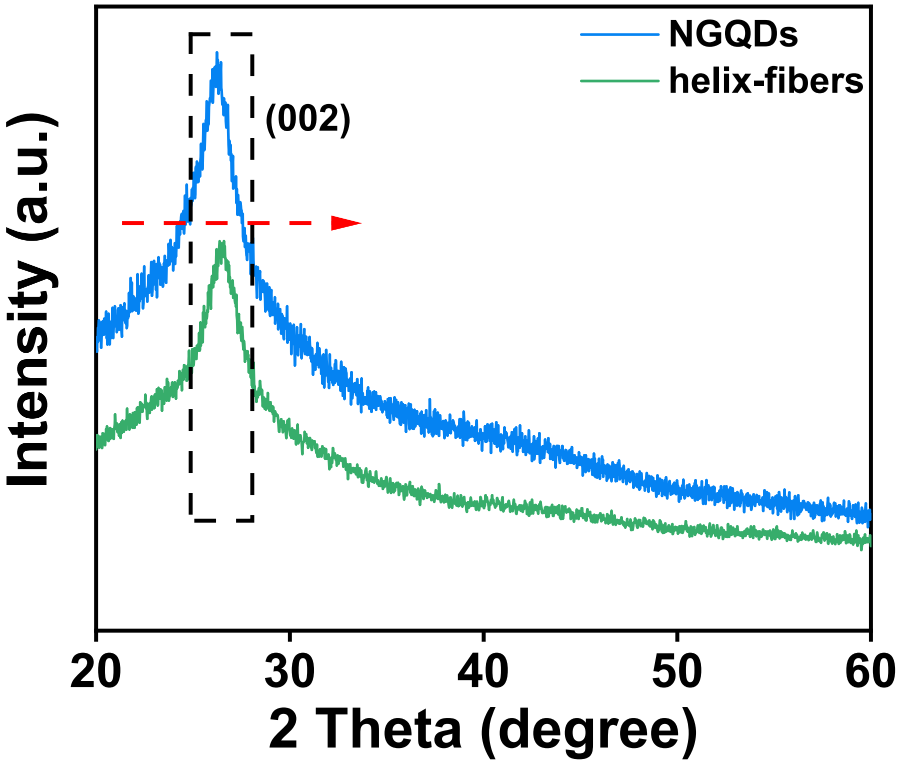

Supplementary Fig. 4: XRD characterization. XRD patterns of NGQDs and helical fibers.

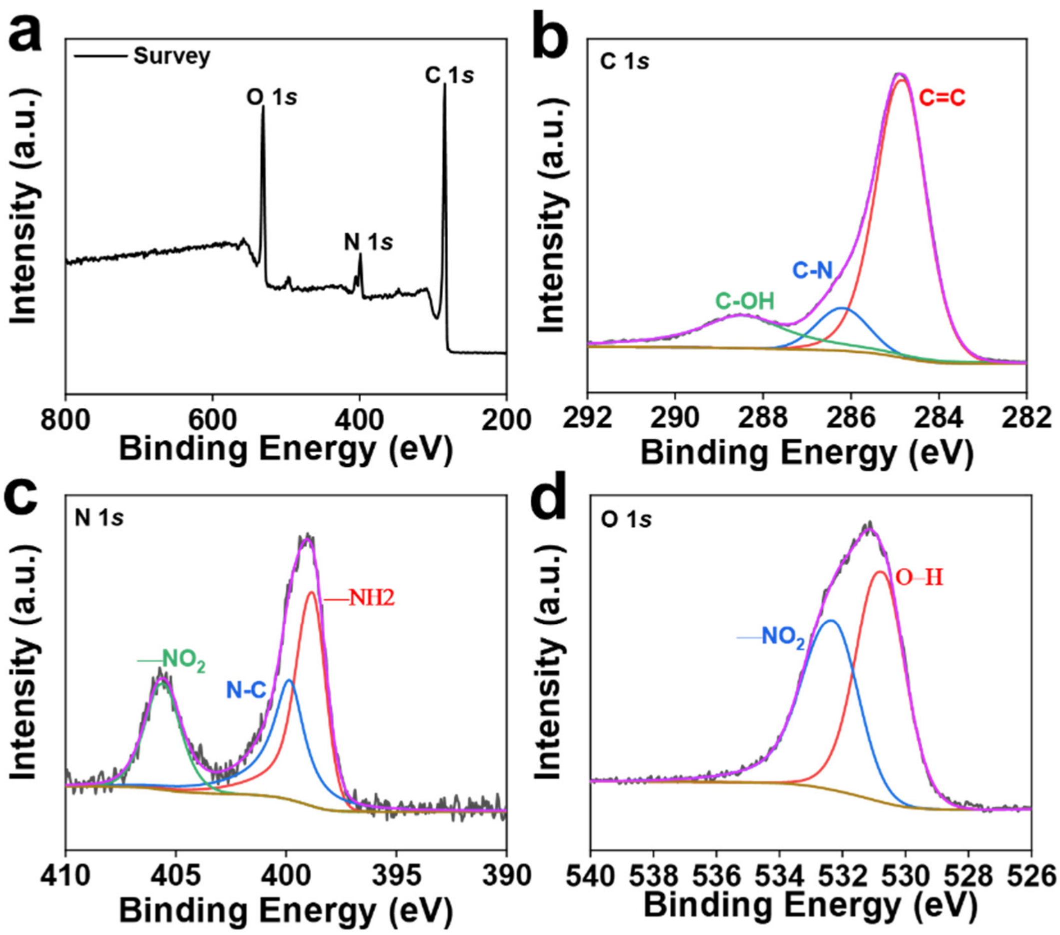

Supplementary Fig. 5: XPS analysis of helical fibers. (**a**) XPS survey spectrum, (**b**) C 1*s*, (**c**) N 1*s*, (**d**) O 1*s* spectra of the helical fibers. Deconvolution of the C 1*s* spectra shows peaks corresponding to C–C/C=C, C–N, and C–OH at 284.8, 286.2, and 288.5 eV, respectively. The N 1*s* spectra show three peaks near 398.8, 399.8, and 405.6 eV, attributed to $–NH_2$, C–N, and $–NO_2$, respectively[2].

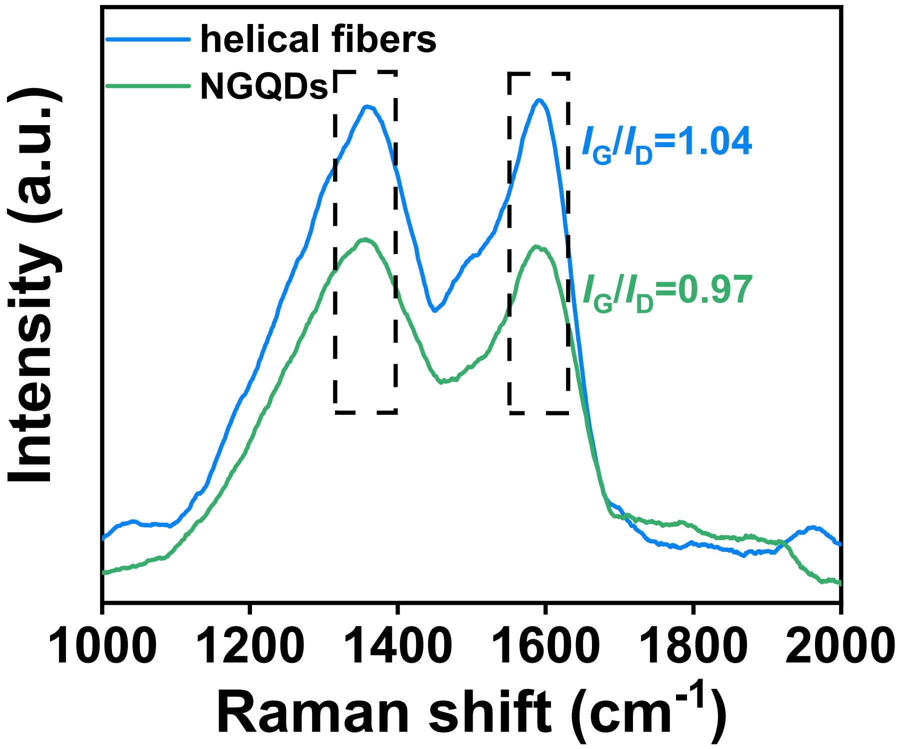

Supplementary Fig. 6: Raman spectra of NGQDs and helical fibers. Raman spectra were employed to demonstrate the presence of π-π interactions within the assembly. The peak at 1596 cm^{-1} (*G*) corresponds to the stretching/shrinking vibration of planar C=C/C–C coupled to the C=O antisymmetric stretching, and the peak at 1354 cm^{-1} (*D*) is associated with the C–H in plane bending. Notably, the former peak is sensitive to π-π stacking interactions, whereas the latter is not[3]. Consequently, the *G*/*D* intensity ratio can be used to evaluate the extent of π-π stacking interaction[4,5]. This suggests that the assembly of NGQDs improves the regularity of coplanar π-π stacking by efficiently packing the aromatic domains, thereby enhancing long-range π-electron delocalization and electron coupling effects[6], and contributing to the uniform loading of metal species.

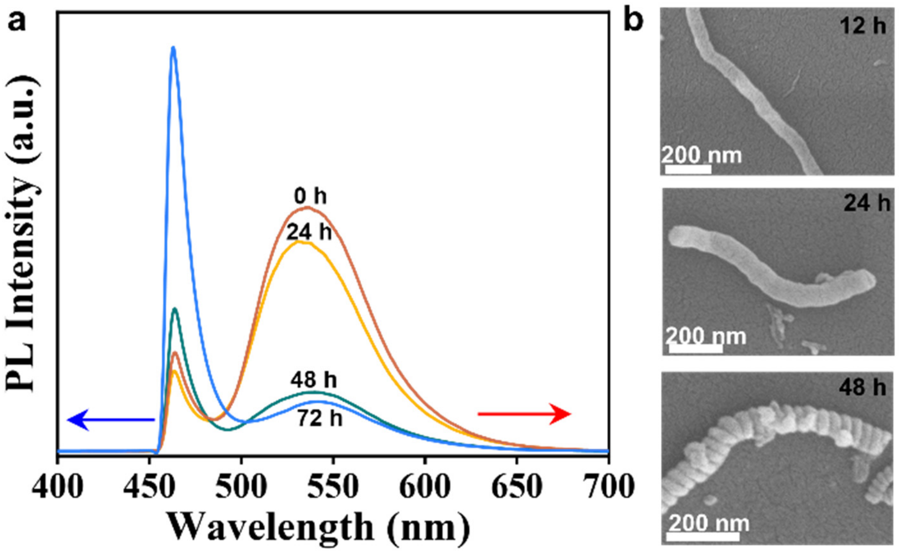

Supplementary Fig. 7: Time-dependent PL spectra and SEM images during the self-assembly of NGQDs. (a) PL spectra, (b) SEM images during the self-assembly of NGQDs into helix-fibers at pH=9.5.

We repeated the self-assembly of NGQDs at pH = 9.5 and collected time-dependent PL data (**Supplementary Fig. 7a**), which showed similar result as that in Fig. 1h.

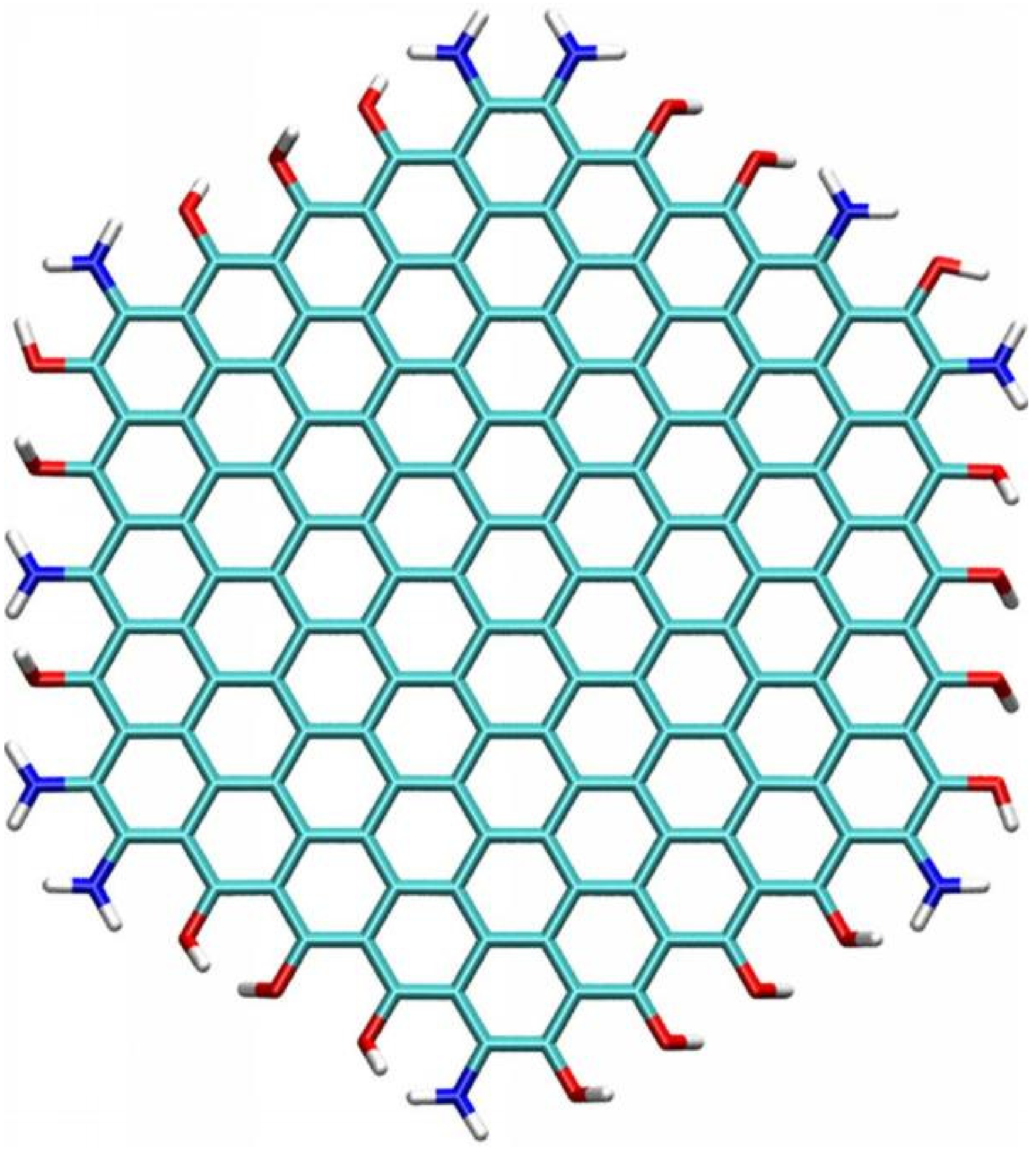

Supplementary Fig. 8: Molecular model of NGQD with hydroxyl and amino groups on the edge. The horizontal and vertical dimensions are 2.3 and 2.6 nm, respectively. Color scheme: green C, blue N, red O and white H. The number of –OH and $–NH_2$ on NGQD molecule can be estimated by XPS analysis. The area occupied by a carbon atom in graphite can be calculated as $(3^{3/2}/4)\times l^2$, where l is the C–C bond length (0.142 nm)[7]. Assuming that NGQD has a quasi-square shape (2.3×2.6 nm^2), the number of carbon atoms in NGQD was estimated to be approximately 203 with 30 peripheral C atoms. XPS analysis gives the atomic ratios of C, N and O are 70.58%, 9.6% and 19.81%. So the ratio of –OH and $–NH_2$ could be calculated from the peak areas in O 1s and N 1s spectra, which is further normalized by $–NO_2$ existed in both spectra. The ratio of $–NH_2$ to –OH is estimated to be 0.54 in NGQD-3, corresponding to the attachment of 10 $–NH_2$ and 20 –OH to the peripheral C.

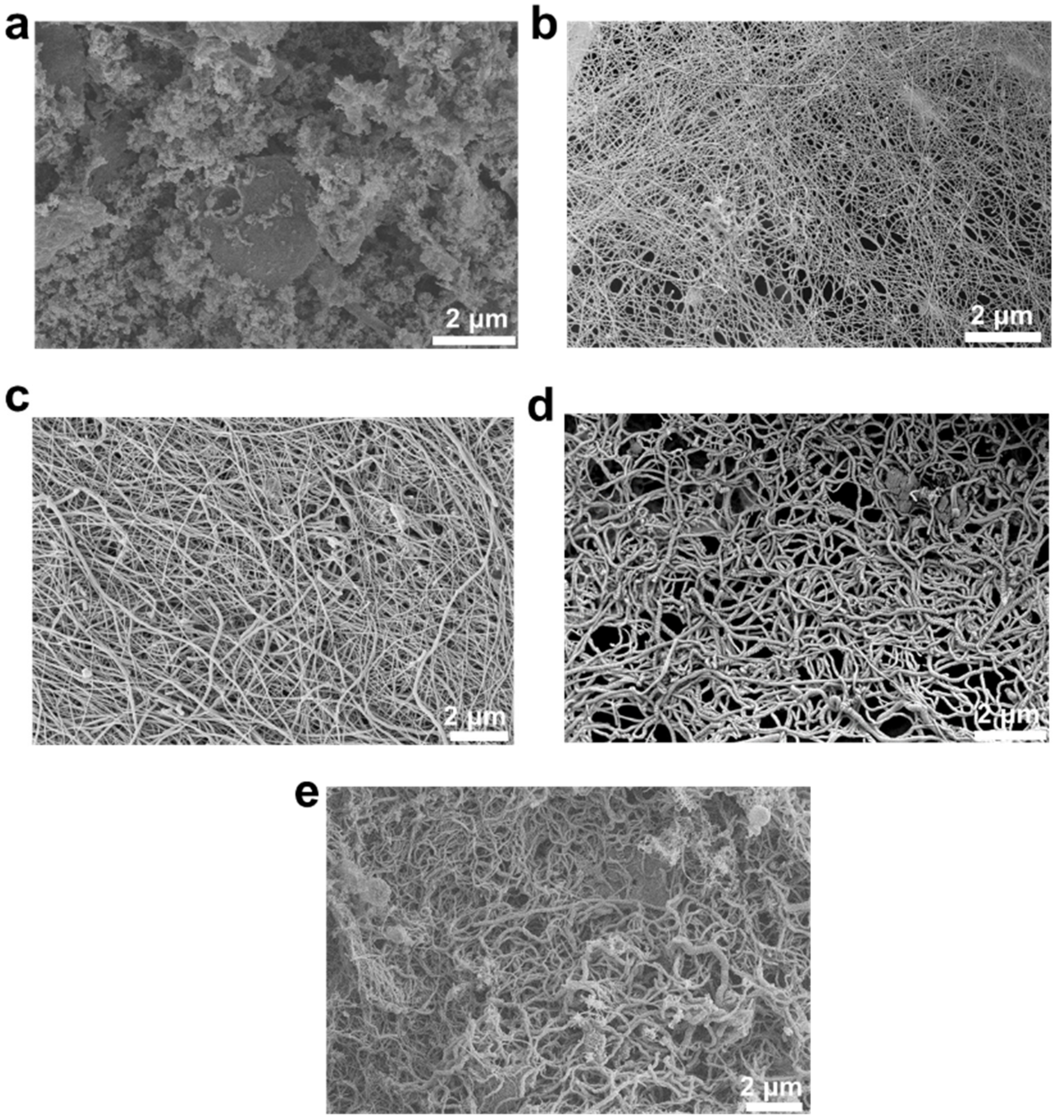

Supplementary Fig. 9: SEM images of the assemblies of NGQDs with varied A/H. (a) 0.69, (b) 0.66, (c) 0.61, (d) 0.54, and (e) 0.43.

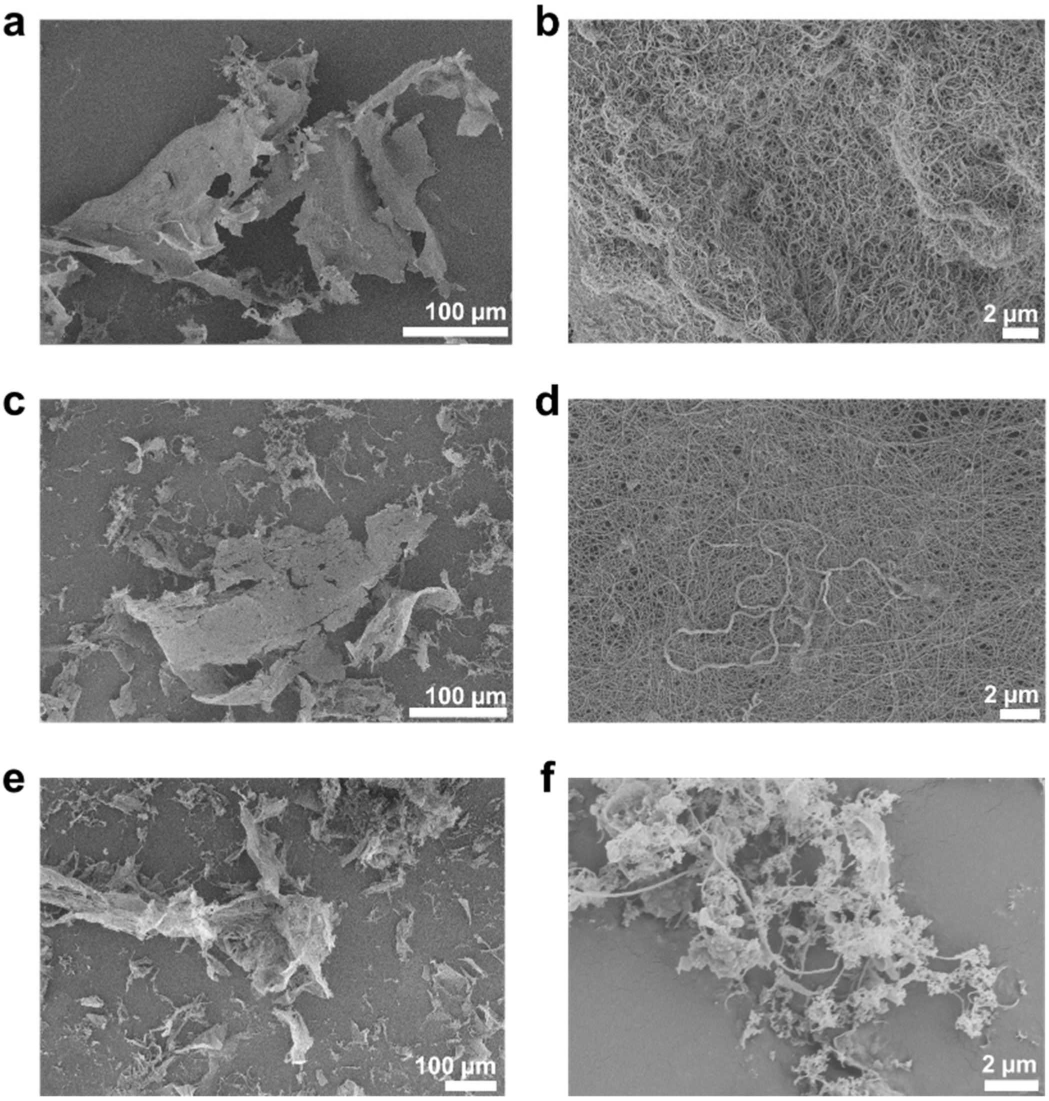

Supplementary Fig. 10: SEM images of the NGQDs assemblies at varied concentration. (a, b) 0.75 mg mL^{-1}, (c, d) 0.375 mg mL^{-1} and (e, f) 0.1875 mg mL^{-1}.

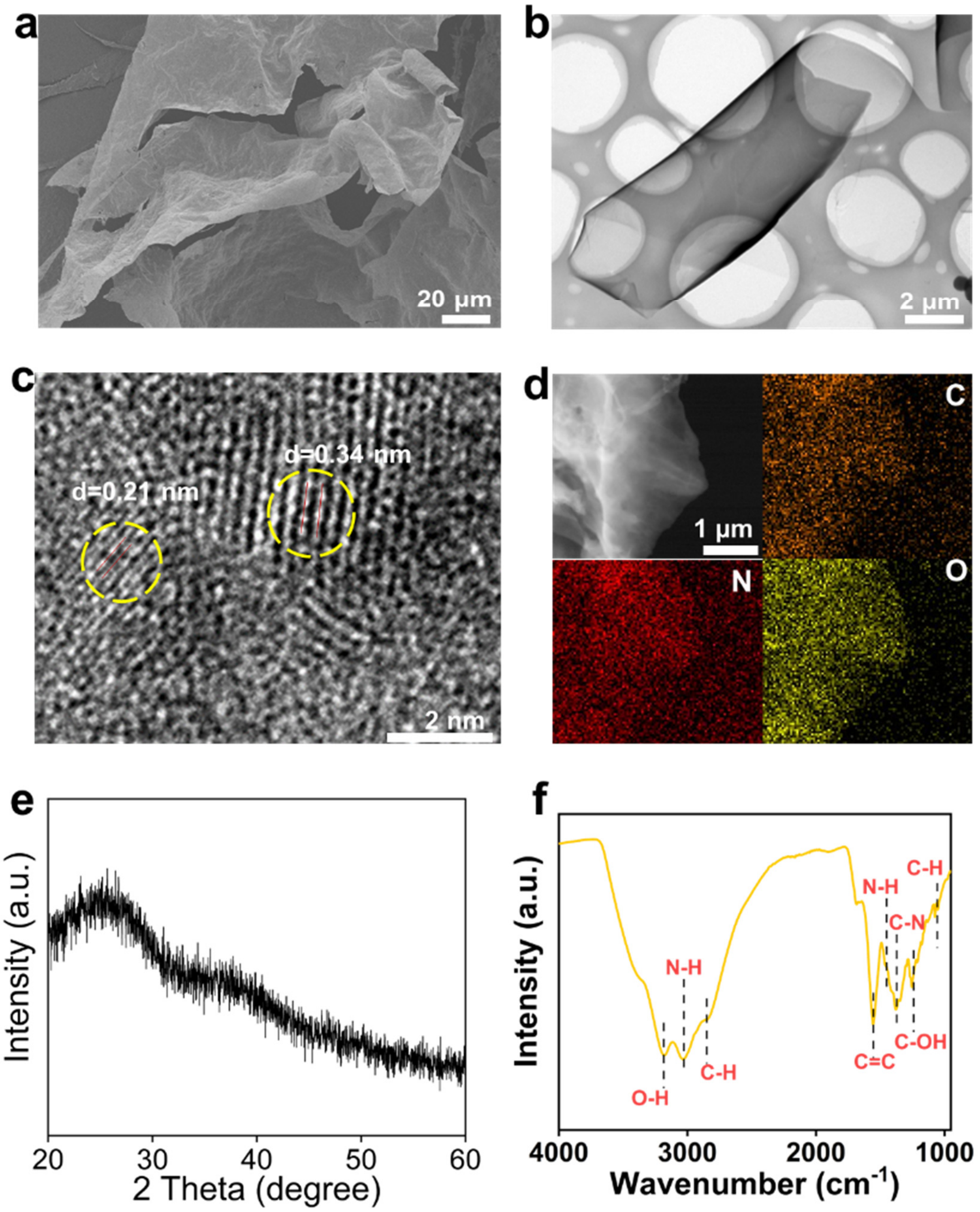

Supplementary Fig. 11: Characterizations of the assembled nanosheets at pH 3.5. (**a**) SEM image, (**b**) TEM image, (**c**) HRTEM image, (**d**) STEM-mapping images, (**e**) XRD pattern, (**f**) FT-IR spectrum of the nanosheets.

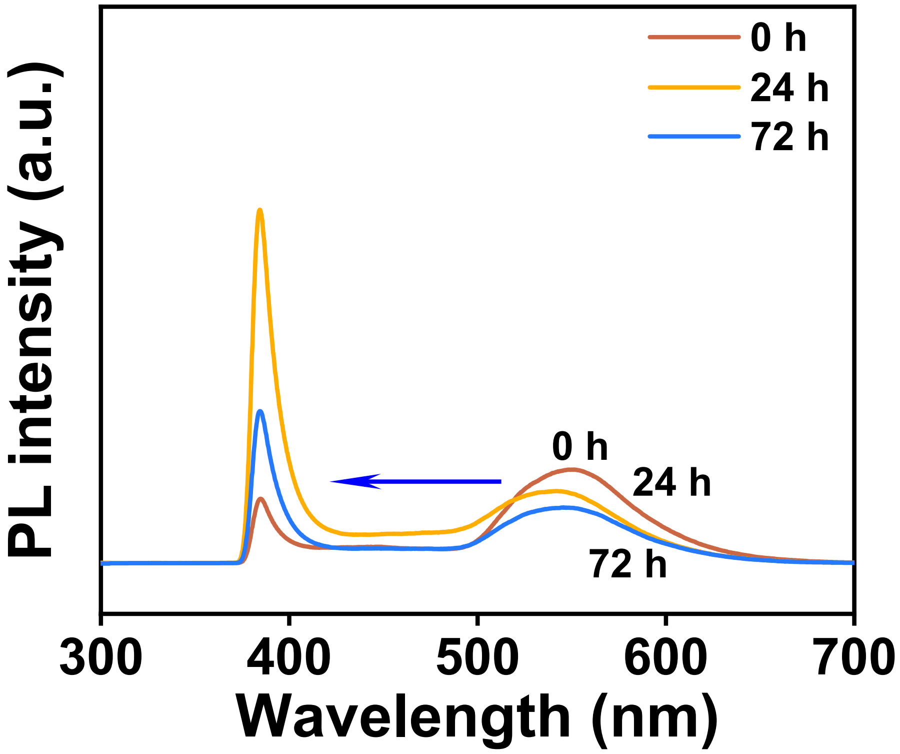

Supplementary Fig. 12: PL spectra during the self-assembly of NGQDs into nanosheets at pH=3.5. PL spectra of the assembly into nanosheets exhibit an obvious blue shift for both band A and band B as function of time, which could be associated with the formation of J-aggregate[8].

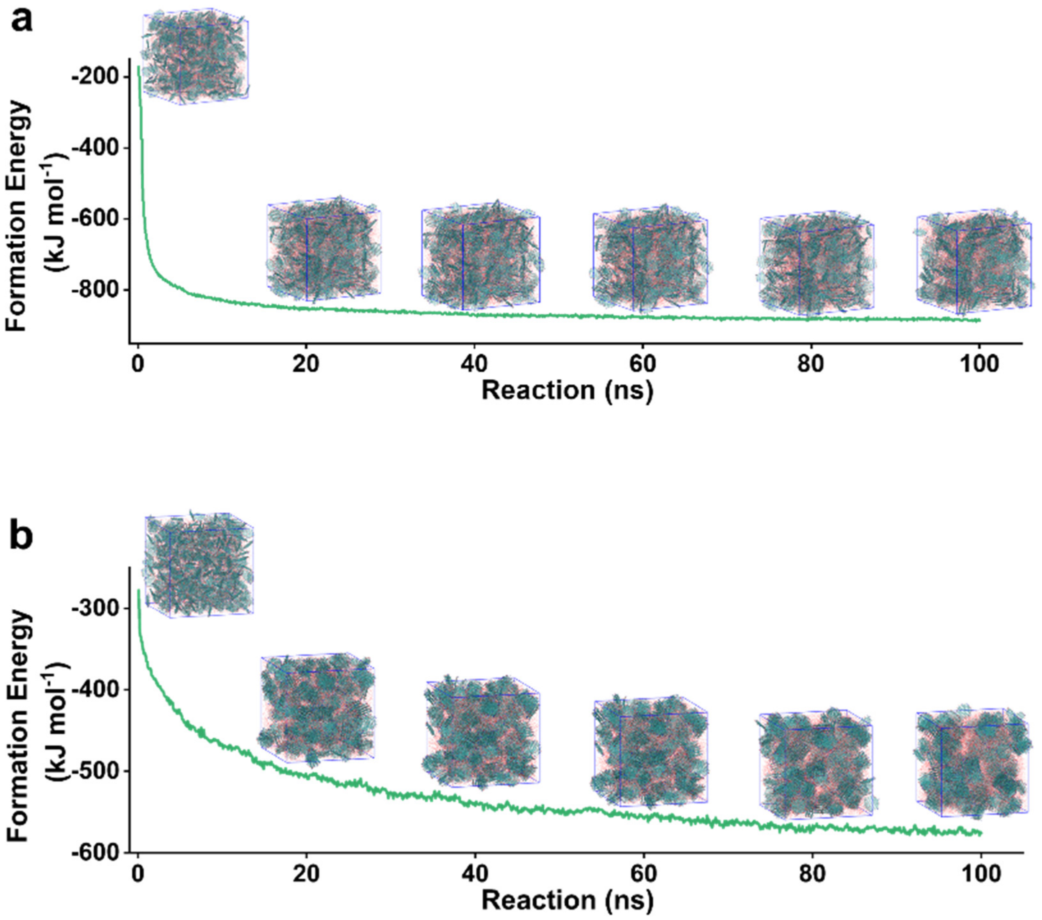

Supplementary Fig. 13: Formation energy and snapshots of self-assembly during 100 ns simulation period. (a) pH=9.5 for helical fibers and (b) pH=3.5 for nanosheets.

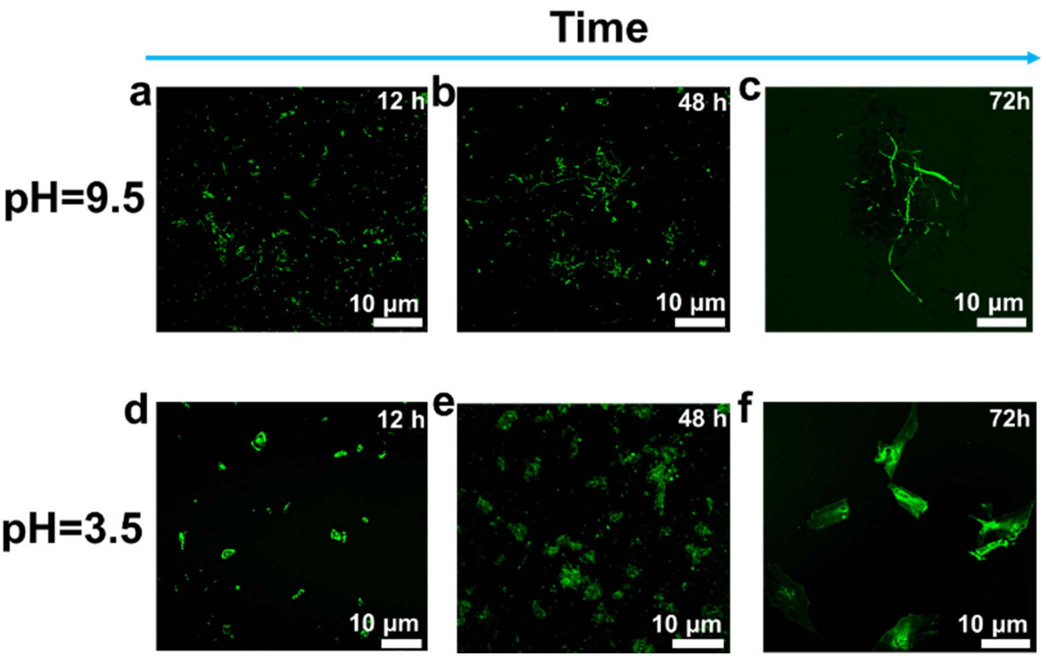

Supplementary Fig. 14: Confocal fluorescence images at different stage of the assembly process. pH=9.5 for helical fibers (a-c) and pH=3.5 for nanosheets (d-f).

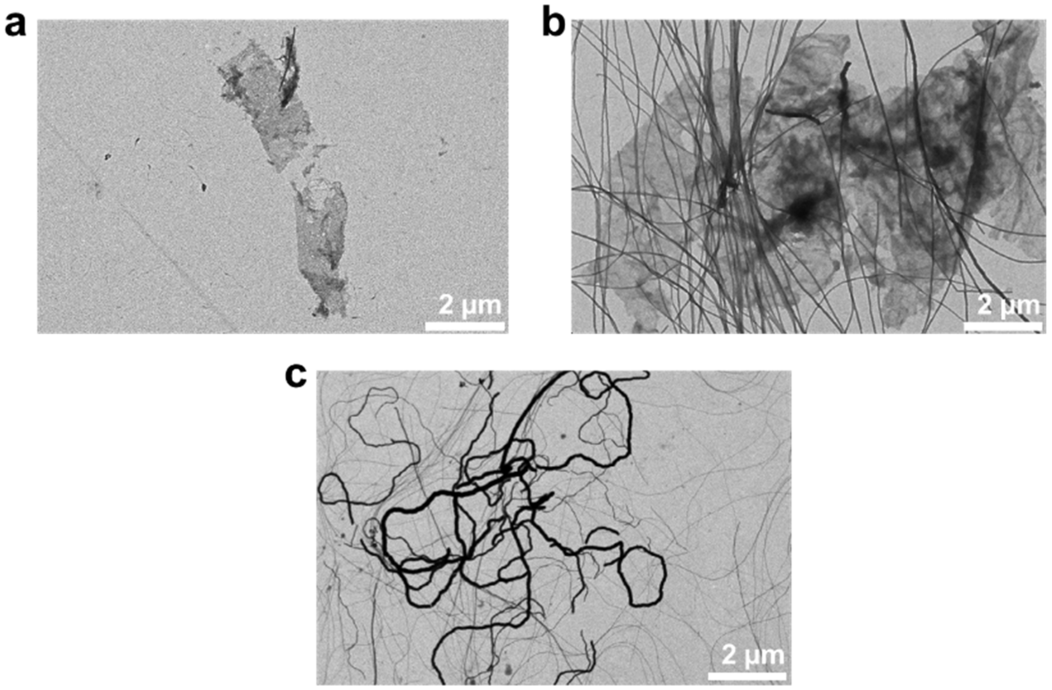

Supplementary Fig. 15: In-situ conversion of nanosheets into fibers via pH modulation. Corresponding TEM images at various stages within the pH change: (**a**) NGQDs assemblies after 72 h incubation at pH 3.5 to form sheets, (**b**) the sheets convert into mixture of fibers and sheets after further 15 h incubation at pH 6.0, (**c**) the mixture converts into absolute fibers after further 20 h incubation at pH 9.5.

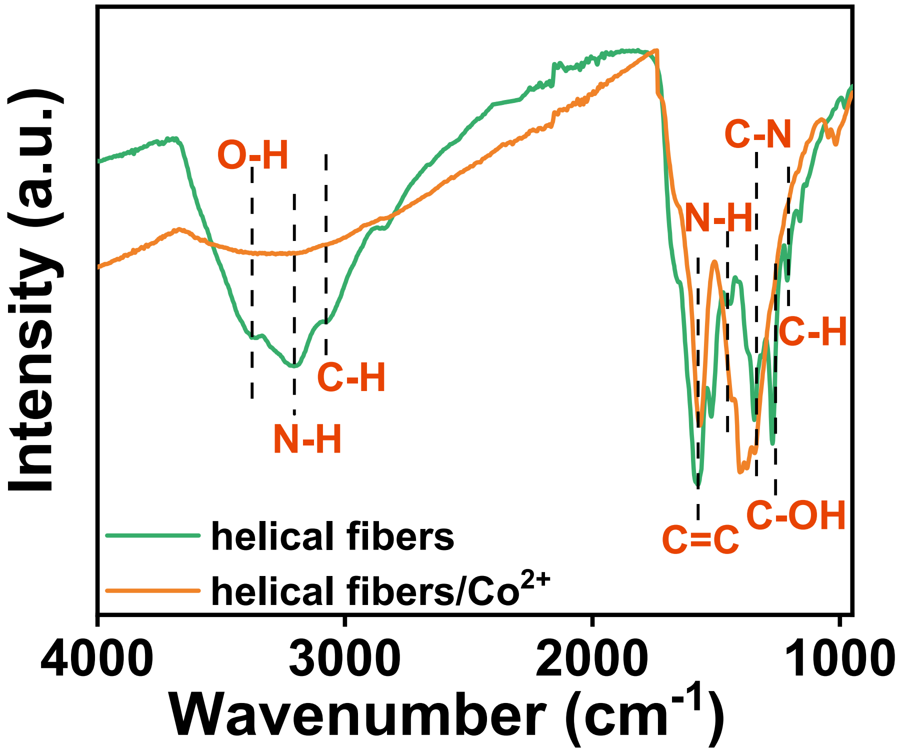

Supplementary Fig. 16: FT-IR spectra. FT-IR spectra of helical fibers and helical fibers/Co^{2+}.

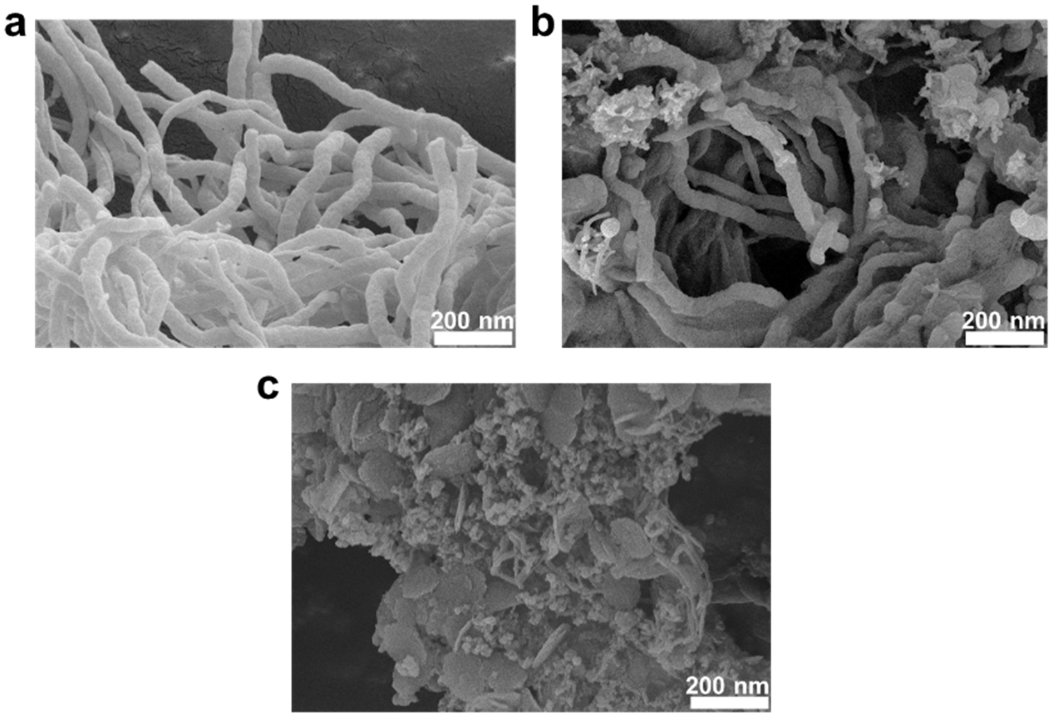

Supplementary Fig. 17: SEM images of Co-PN-C obtained at different temperature. (a) Co–PN–C_{500}, (b) Co–PN–C_{700}, and (c) Co–PN–C_{800}.

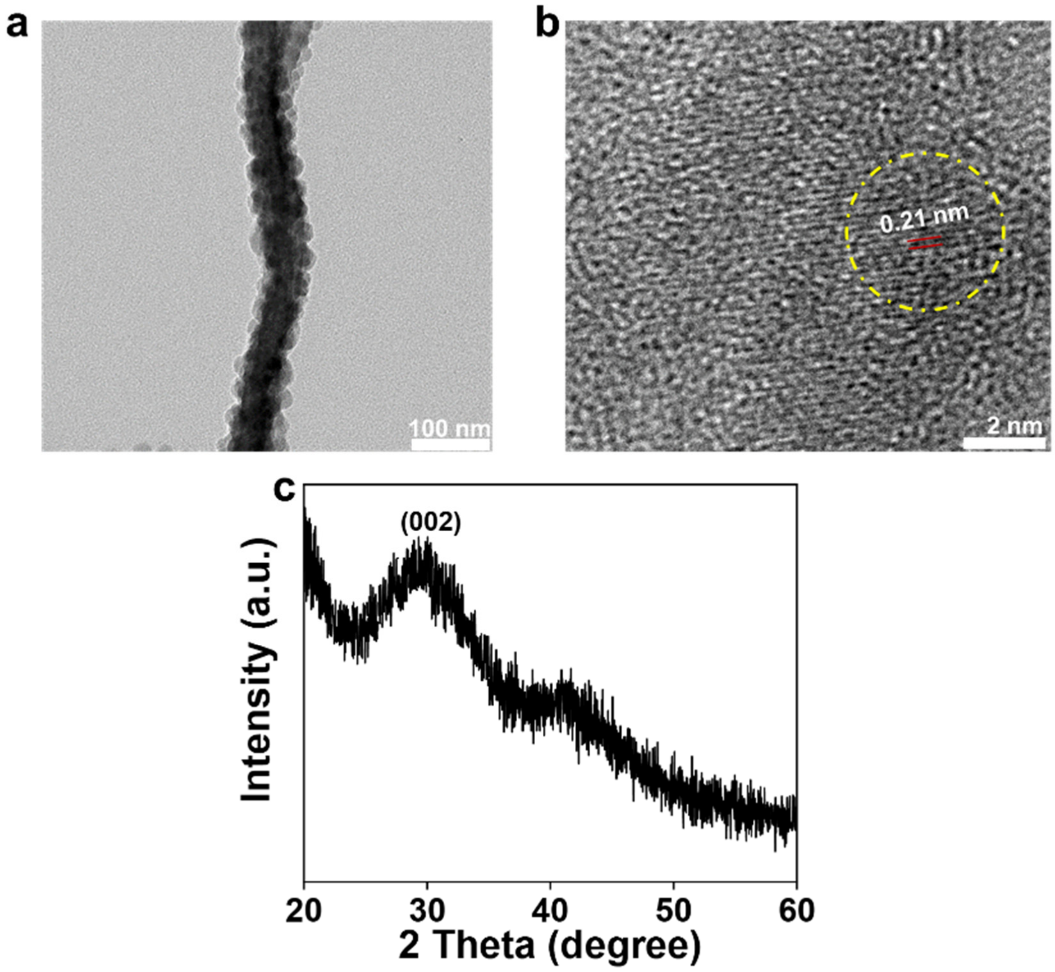

Supplementary Fig. 18: Structure characterizations of Co–PN–C_{500}. (a) TEM image, (b) HRTEM image, and (c) XRD pattern of Co–PN–C_{500}.

The characteristic diffraction peaks at 29.4° and 41.6° correspond to the graphitic carbon.

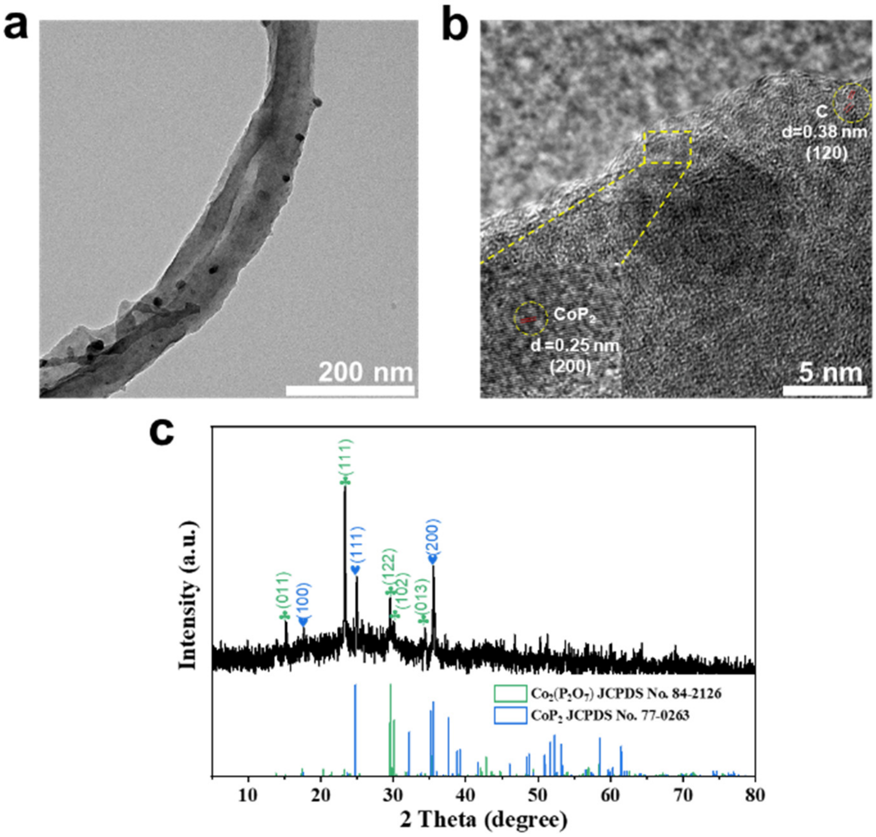

Supplementary Fig. 19: Characterizations of Co–PN–C_{700}. (**a**) TEM image, (**b**) HRTEM image, (**c**) XRD pattern of Co–PN–C_{700}.

The characteristic diffraction peaks at 15.2°, 23.3°, 29.5°, 30.2°, 34.3° are assigned to (011), (111), (122), (102) and (013) planes of $Co_2(P_2O_7)$ (JCPDS No. 84-2126), and the ones located at 17.6°, 24.6°, 35.6° are assigned to (100), (111) and (200) planes of CoP_2 (JCPDS No.77-0263), respectively. The strong peak intensity at 23.3° could also origin from the carbon substrate with interplanar spacing of 0.38 nm corresponding to the (120) plane (JCPDS No. 50-0926)[9].

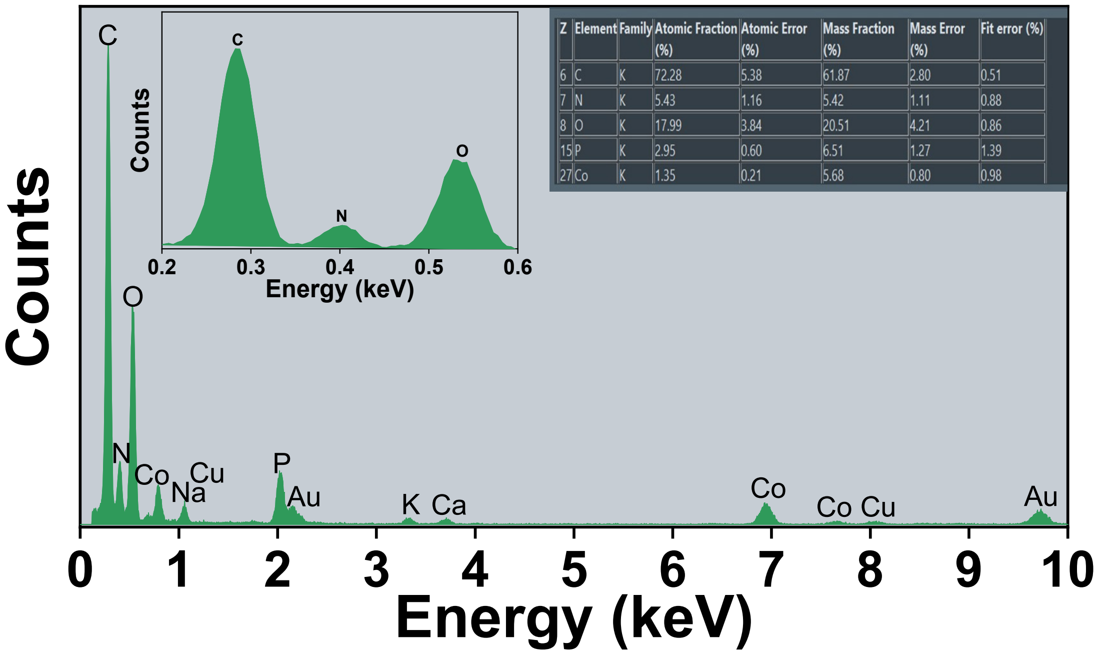

Supplementary Fig. 20: EDS spectrum of the corresponding elemental mapping in Fig. 3d (inset: the enlarged spectrum within the energy range of 0-1 keV and the quantification table). It indicates the rough content of C, Co, N, P and O is around 61.87 wt%, 5.68 wt%, 5.42 wt%, 6.51 wt% and 20.51 wt%, respectively.

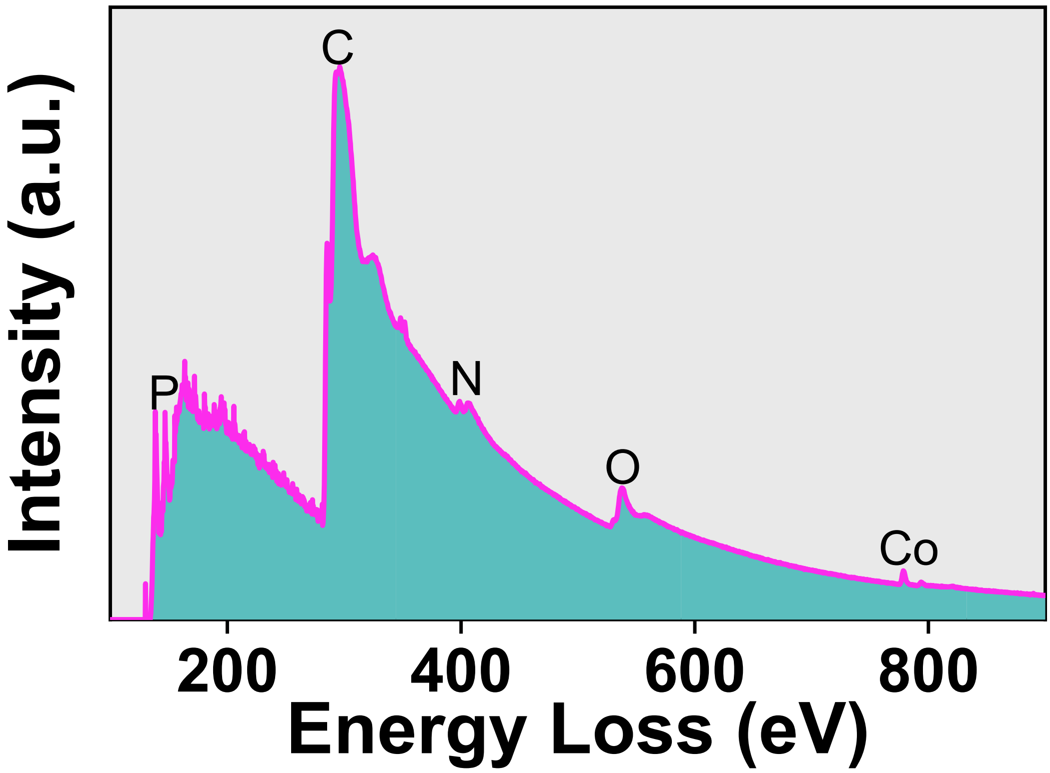

Supplementary Fig. 21: Elemental characterization. The EELS spectrum of Co–PN–C_{600}.

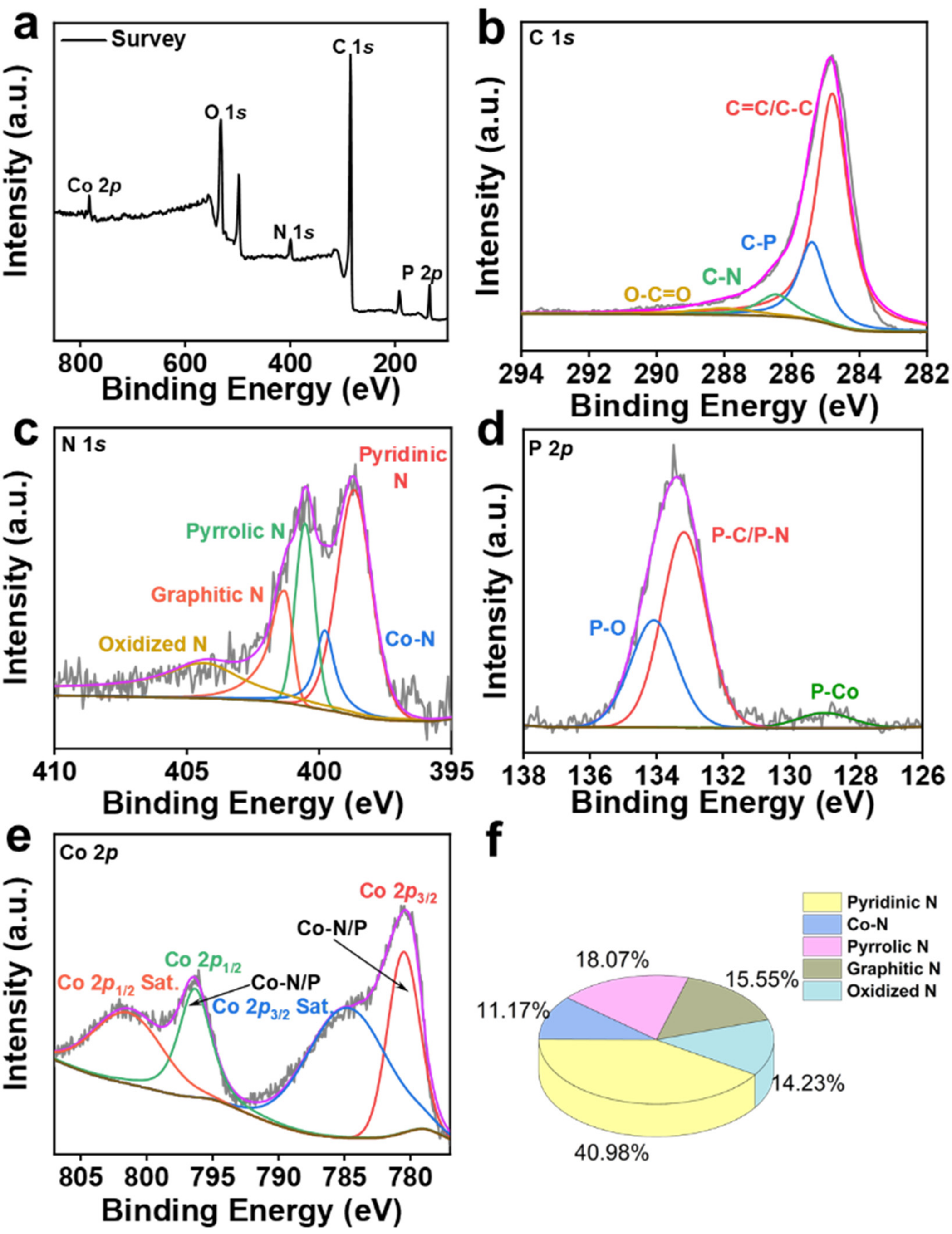

Supplementary Fig. 22: XPS analysis of Co–PN–C$_{600}$. (**a**) XPS survey spectrum, (**b**) C 1*s*, (**c**) N 1*s*, (**d**) P 2*p*, (**e**) Co 2*p* spectra of Co–PN–C$_{600}$. (**f**) Distribution of N element with different bonding configurations.

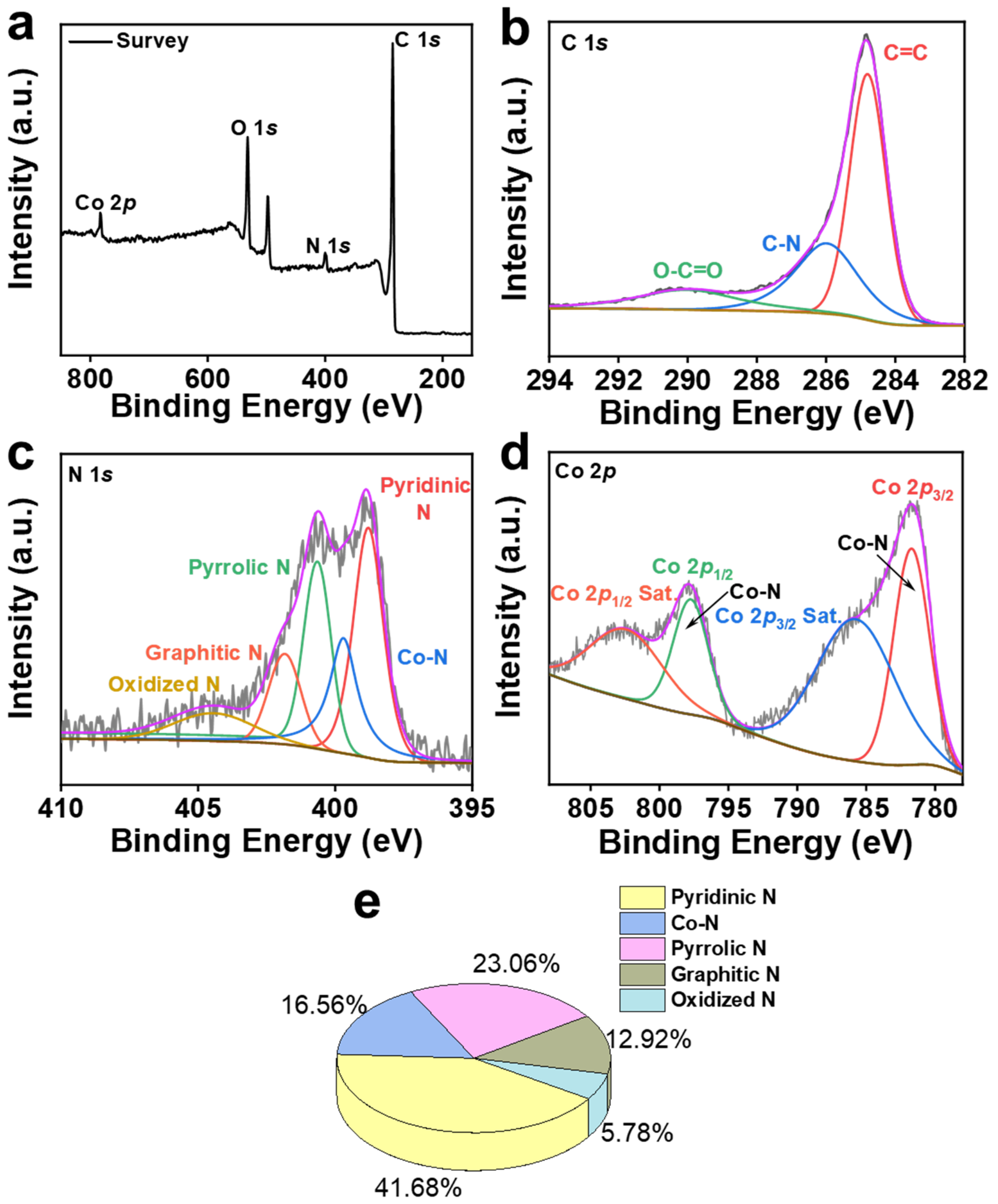

Supplementary Fig. 23: XPS analysis of Co–N4–C. (**a**) XPS survey spectrum, (**b**) C 1*s*, (**c**) N 1*s*, (**d**) Co 2*p* spectra of Co–N4–C. (**e**) Distribution of N element with different bonding configurations.

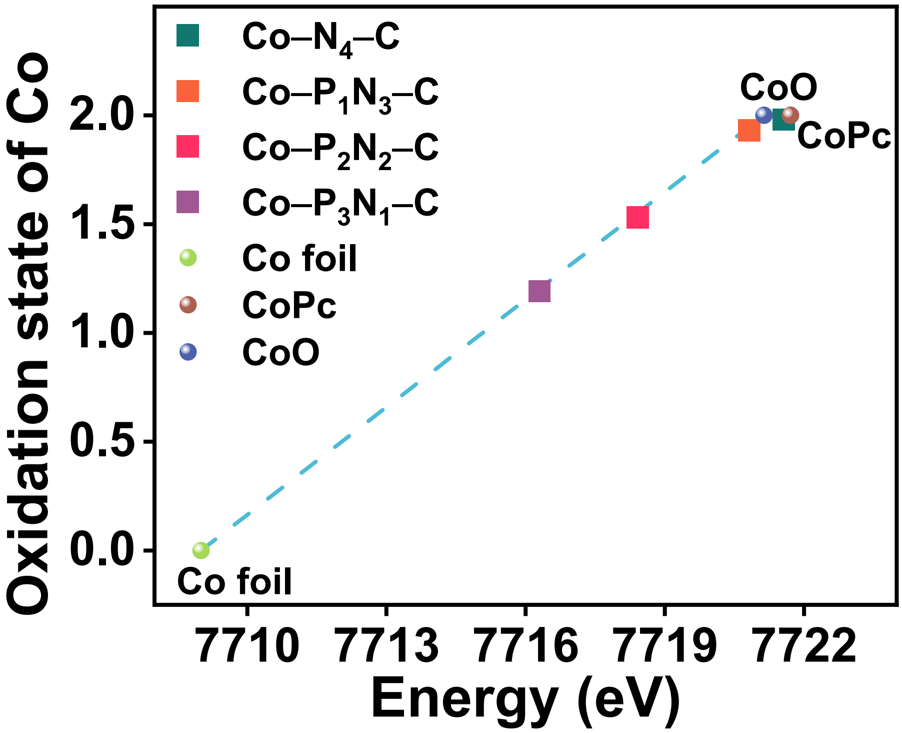

Supplementary Fig. 24: Oxidation states of Co. The fitted average oxidation states of Co from XANES spectra.

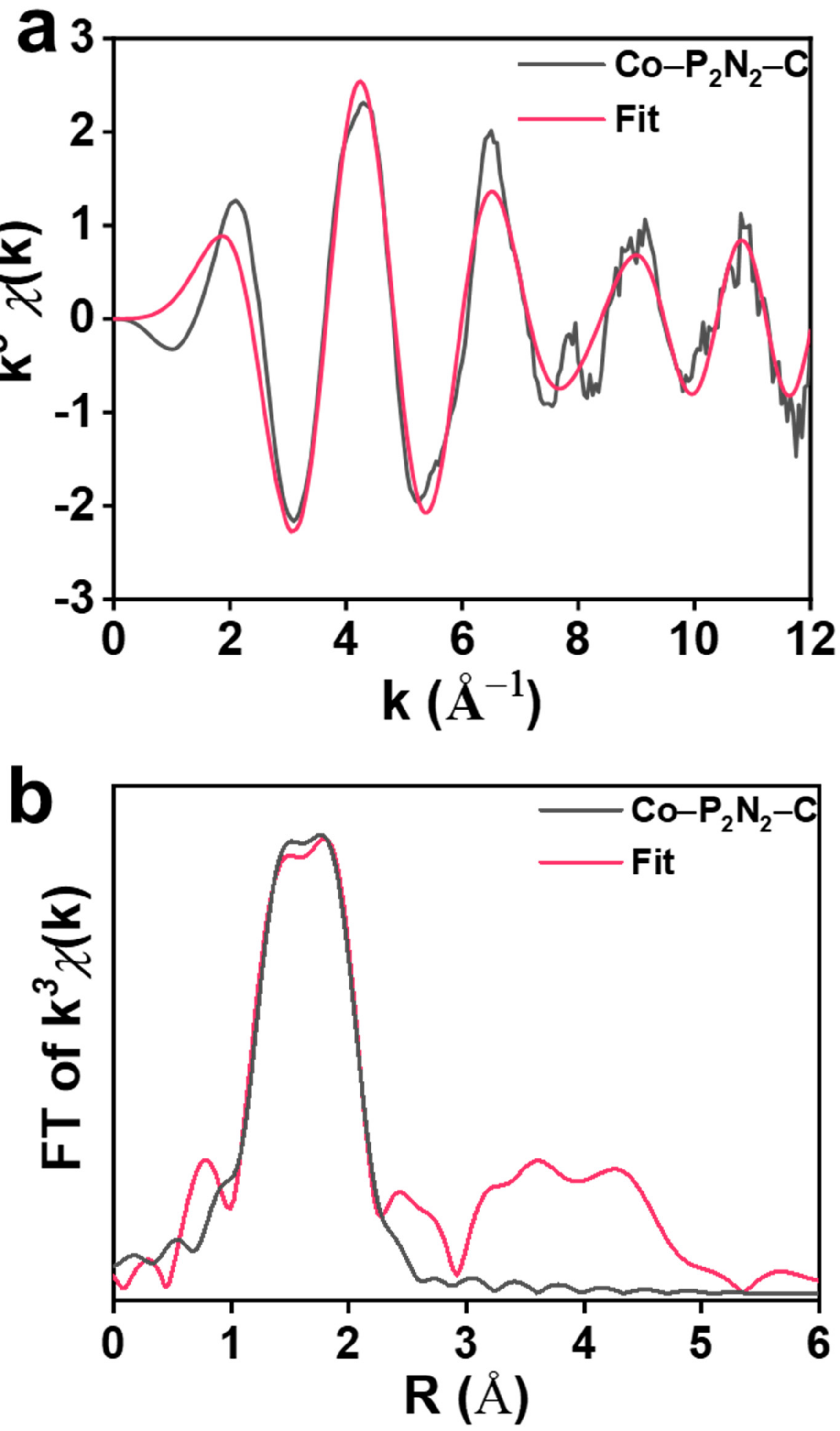

Supplementary Fig. 25: FT-EXAFS fitting curves. FT-EXAFS fitting curves of Co–PN–C_{600} (Co–P_2N_2–C) at (**a**) k space and (**b**) R space.

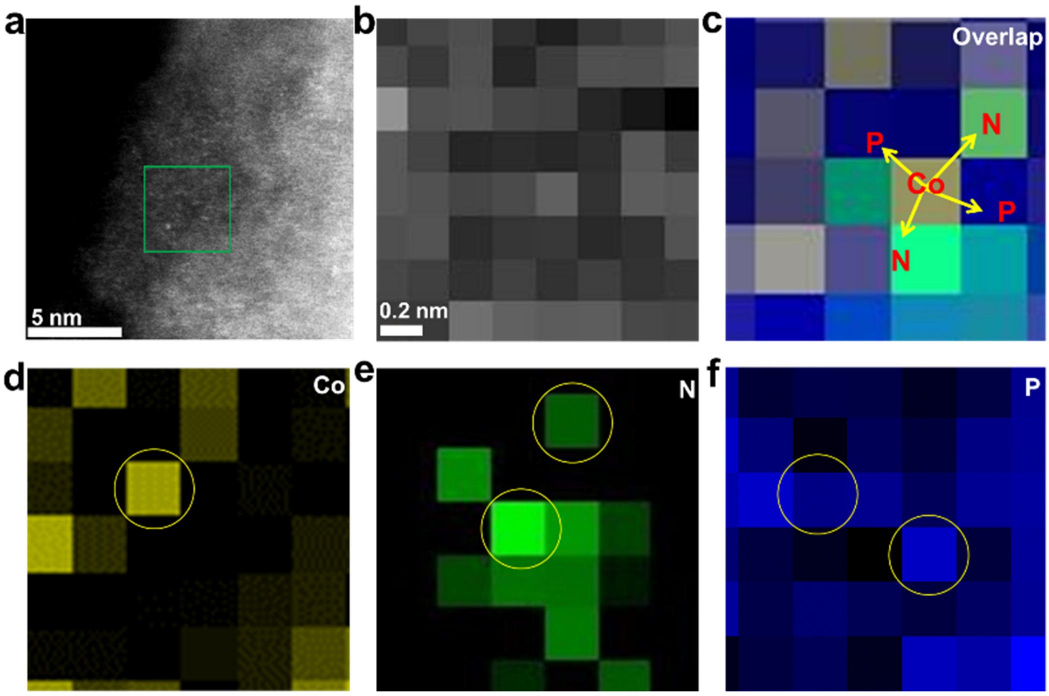

Supplementary Fig. 26: Atomic-resolution EELS analysis of Co–P_2N_2–C. (a) STEM-HAADF image. (b) A synchronous HAADF image acquired with the EELS mapping within the green rectangle. (c) The overlap mapping. Elemental mappings of (d) Co, (e) N, and (f) P.

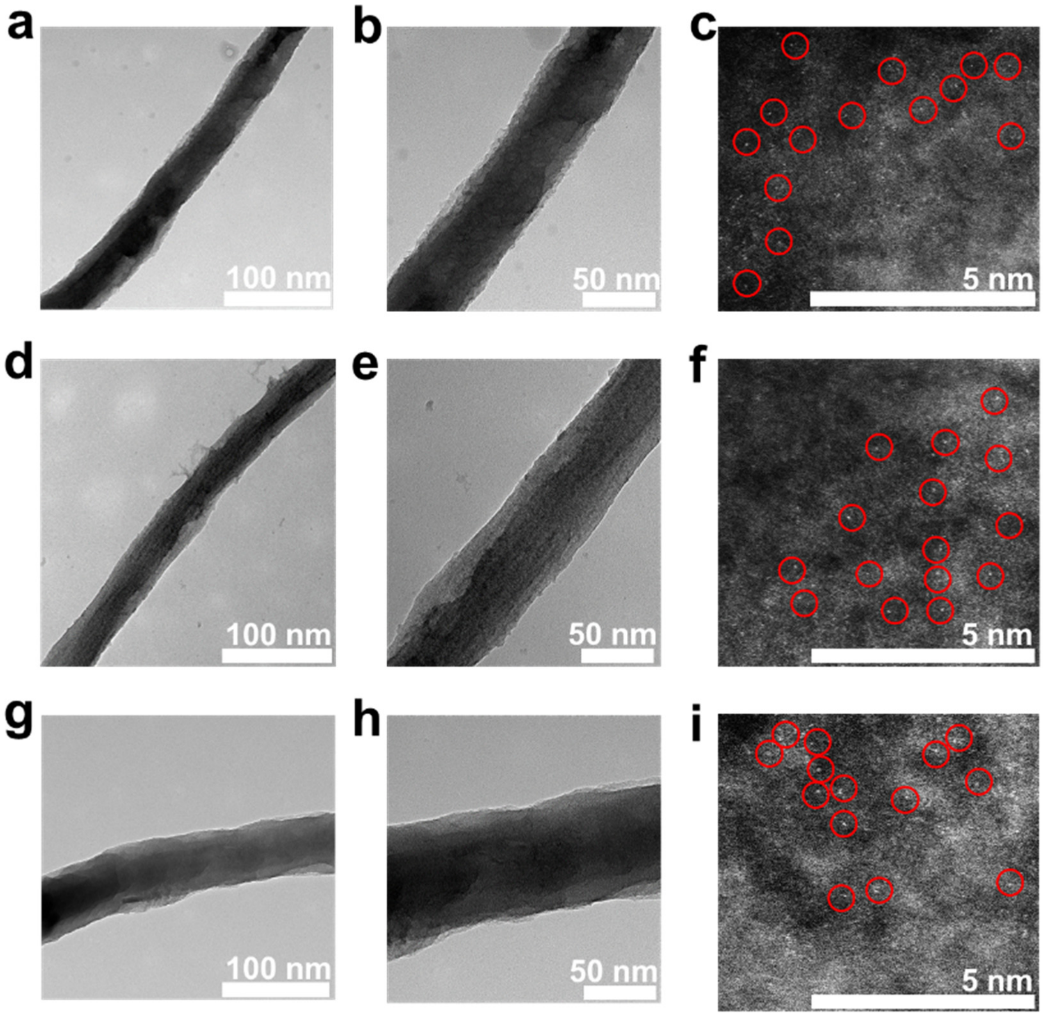

Supplementary Fig. 27: Atomic-scale characterizations of the Co-PN-C. TEM and the high-resolution HAADF-STEM image with spherical aberration correction for (**a-c**) Co–N_4–C, (**d-f**) Co–P_1N_3–C, and (**g-i**) Co–P_3N_1–C.

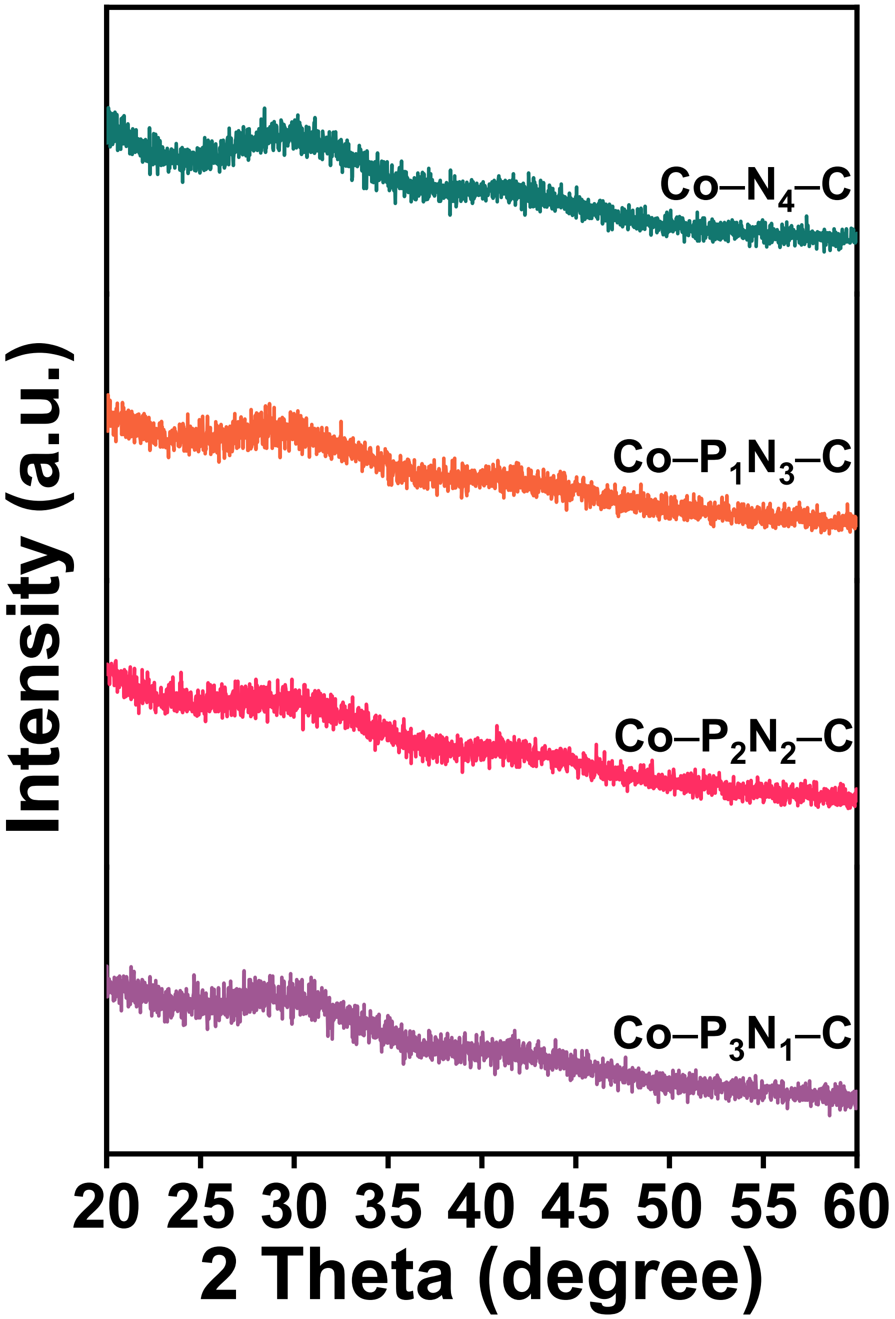

Supplementary Fig. 28: XRD characterizations. XRD patterns of Co–N_4–C, Co–P_1N_3–C, Co–P_2N_2–C and Co–P_3N_1–C.

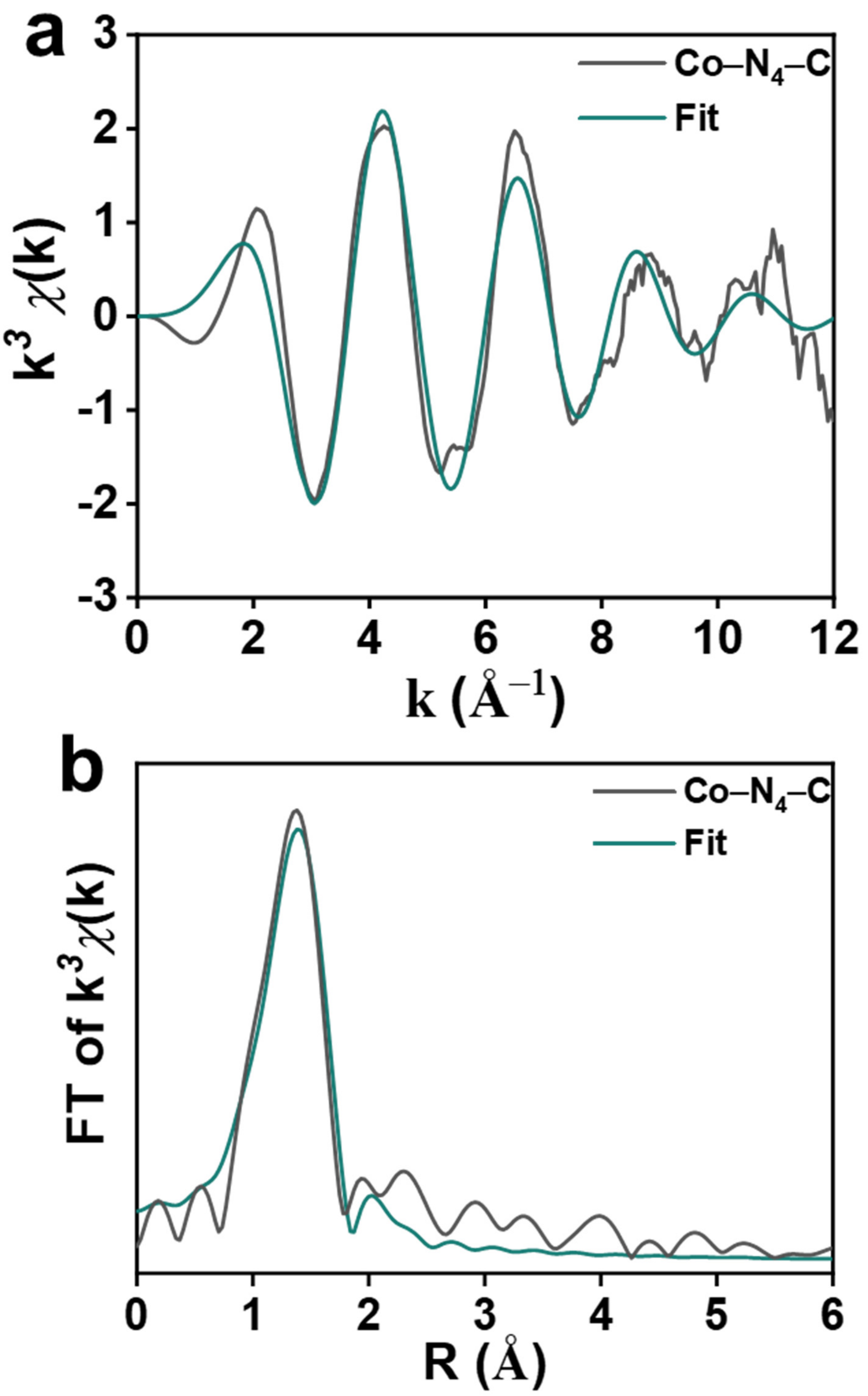

Supplementary Fig. 29: FT-EXAFS fitting curves. FT-EXAFS fitting curves of Co–N_4–C at (**a**) k space and (**b**) R space.

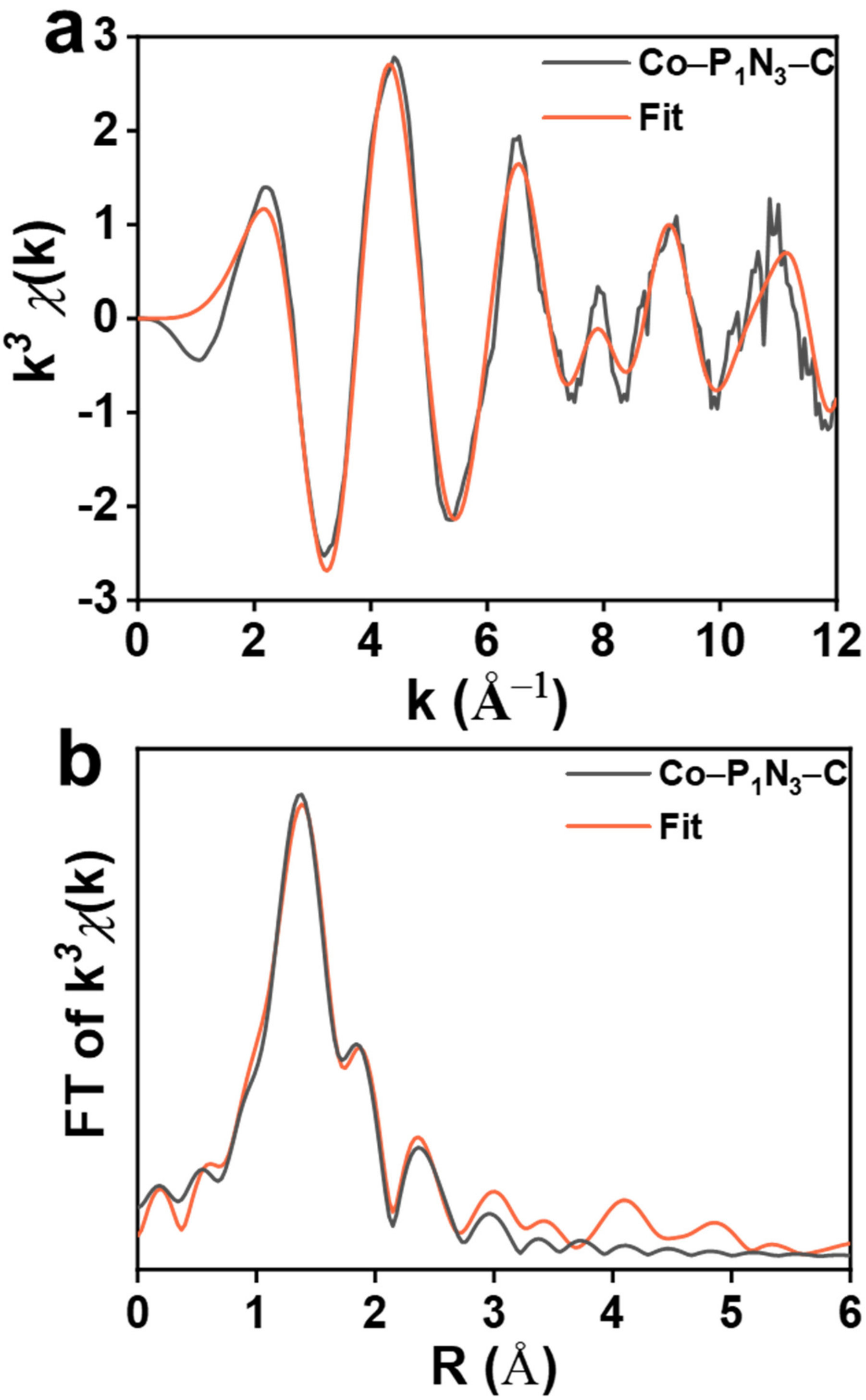

Supplementary Fig. 30: FT-EXAFS fitting curves. FT-EXAFS fitting curves of Co–P_1N_3–C at (**a**) k space and (**b**) R space.

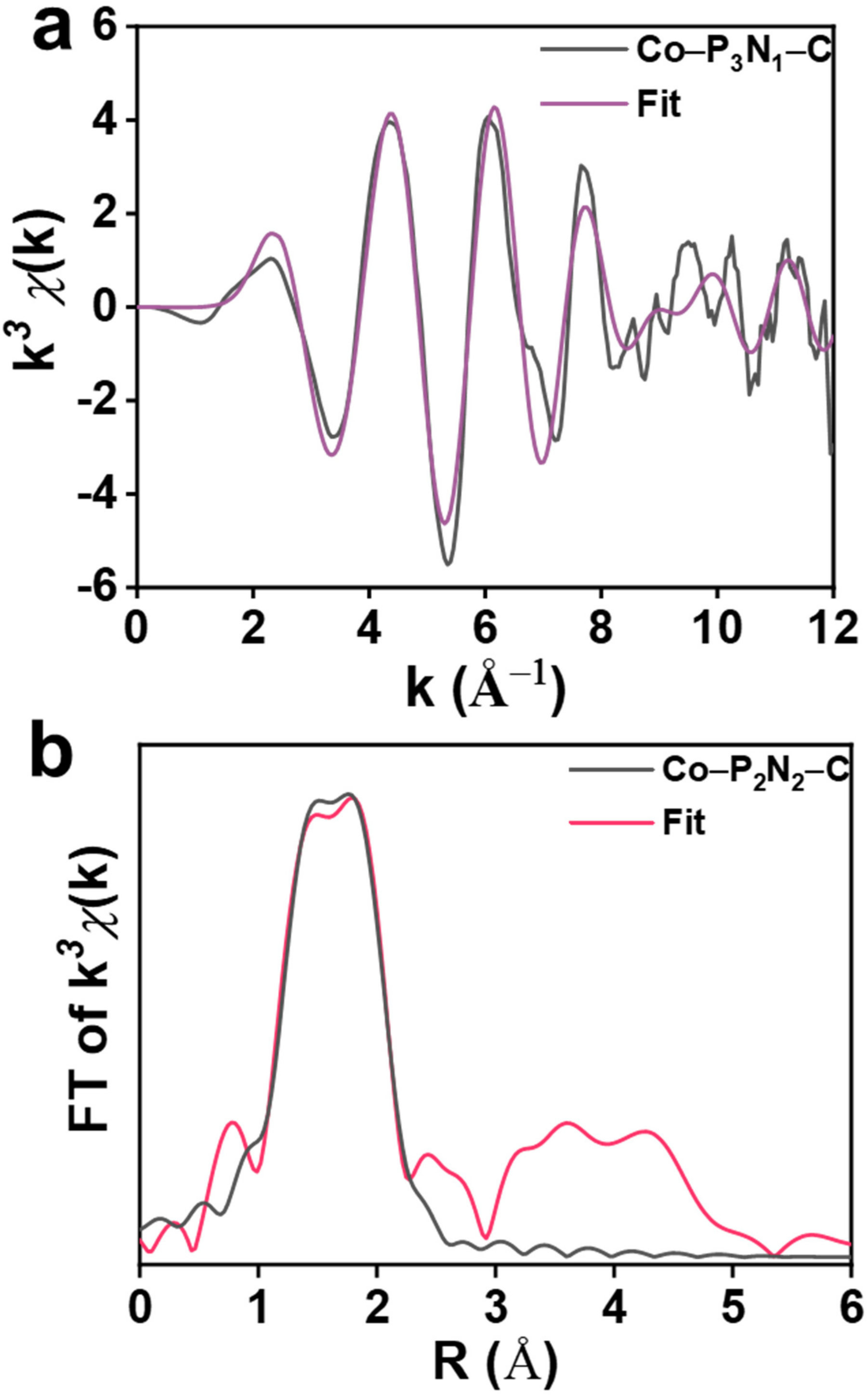

Supplementary Fig. 31: FT-EXAFS fitting curves. FT-EXAFS fitting curves of Co–P_3N_1–C at (**a**) k space and (**b**) R space.

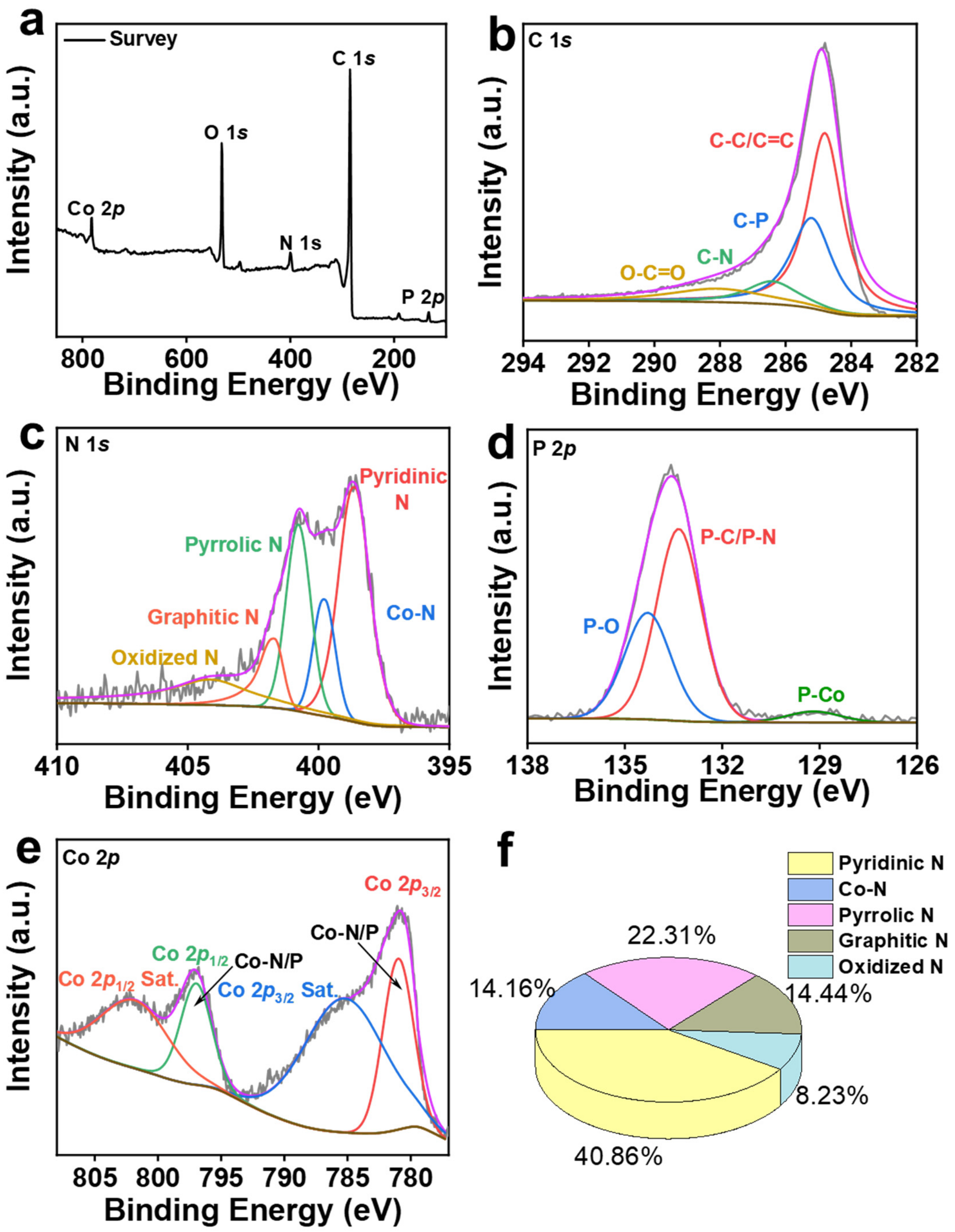

Supplementary Fig. 32: XPS analysis of Co–P$_1$N$_3$–C. (**a**) XPS survey spectrum, (**b**) C 1*s*, (**c**) N 1*s*, (**d**) P 2*p*, (**e**) Co 2*p* spectra of Co–P$_1$N$_3$–C. (**f**) Distribution of N element with different bonding configurations.

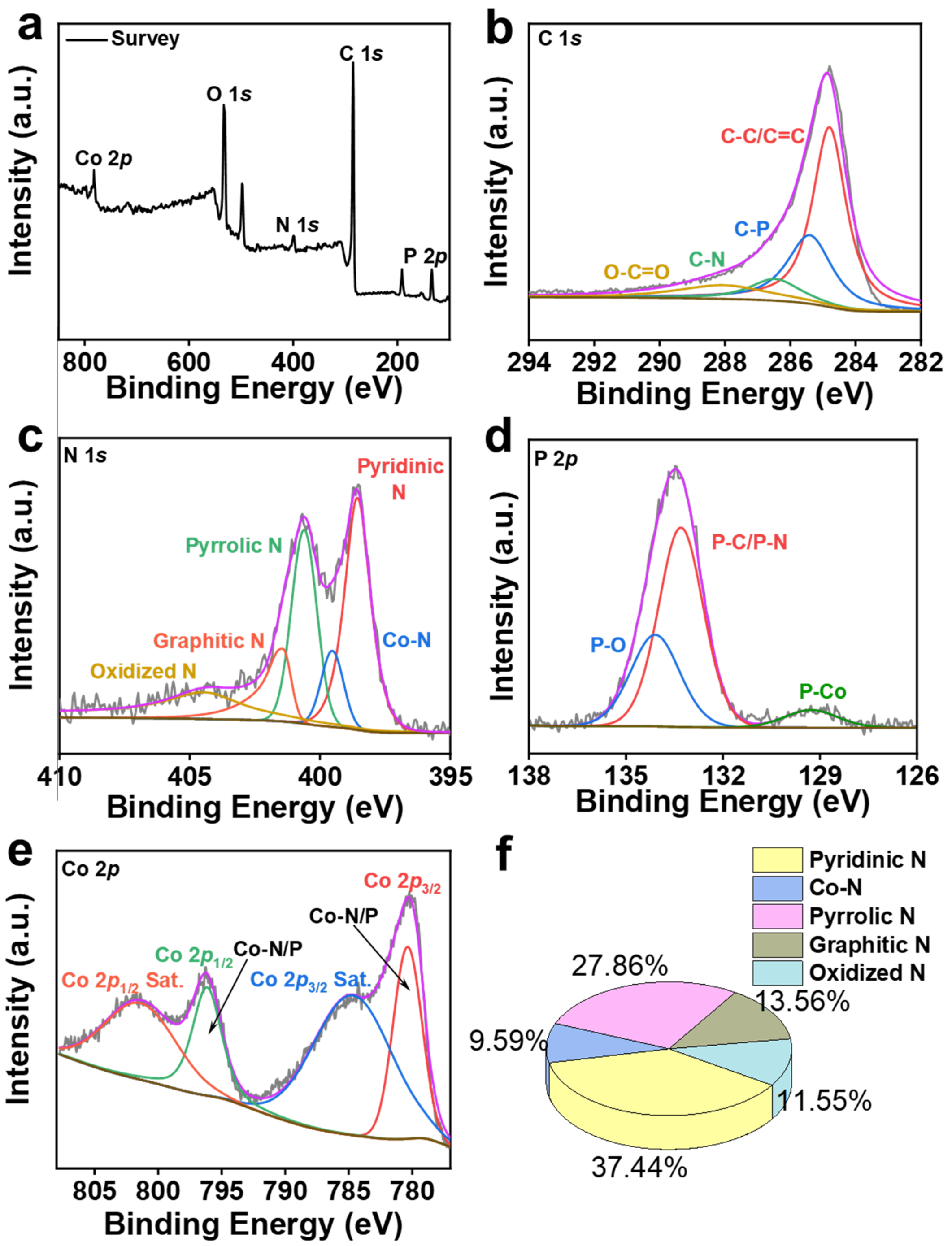

Supplementary Fig. 33: XPS analysis of Co–P_3N_1–C. (**a**) XPS survey spectrum, (**b**) C 1*s*, (**c**) N 1*s*, (**d**) P 2*p*, (**e**) Co 2*p* spectra of Co–P_3N_1–C. (**f**) Distribution of N element with different bonding configurations.

Electrochemical active surface area (ECSA): The capacitive currents were measured in a potential window where no faradic process is observed (0.03-0.13 V vs. RHE). The CVs were collected at various sweep rates from 10 to 200 mV s^{-1}. ECSA were estimated from the as-obtained double-layer capacitance (C_{dl}), which can be calculated following the equations listed below:

$$C_{dl} = \frac{Q}{U} = \frac{dQ/dt}{dU/dt} = \frac{j}{r} \quad (1)$$

where Q is the quantity of electric charge per unit area, U is the voltage (V), j is the current density (mA cm^{-2} mg^{-1}), r is the scan rate (V s^{-1}). Then the ECSA was calculated as:

$$\text{ECSA} = \frac{C_{dl}}{C_s} \quad (2)$$

where C_s is the specific capacitance value of a flat standard with 1 cm^2 of real surface area. The general value for C_s is between 20 μF cm^{-2} and 60 μF cm^{-2}. Here we use the average value (40 μF cm^{-2})[10].

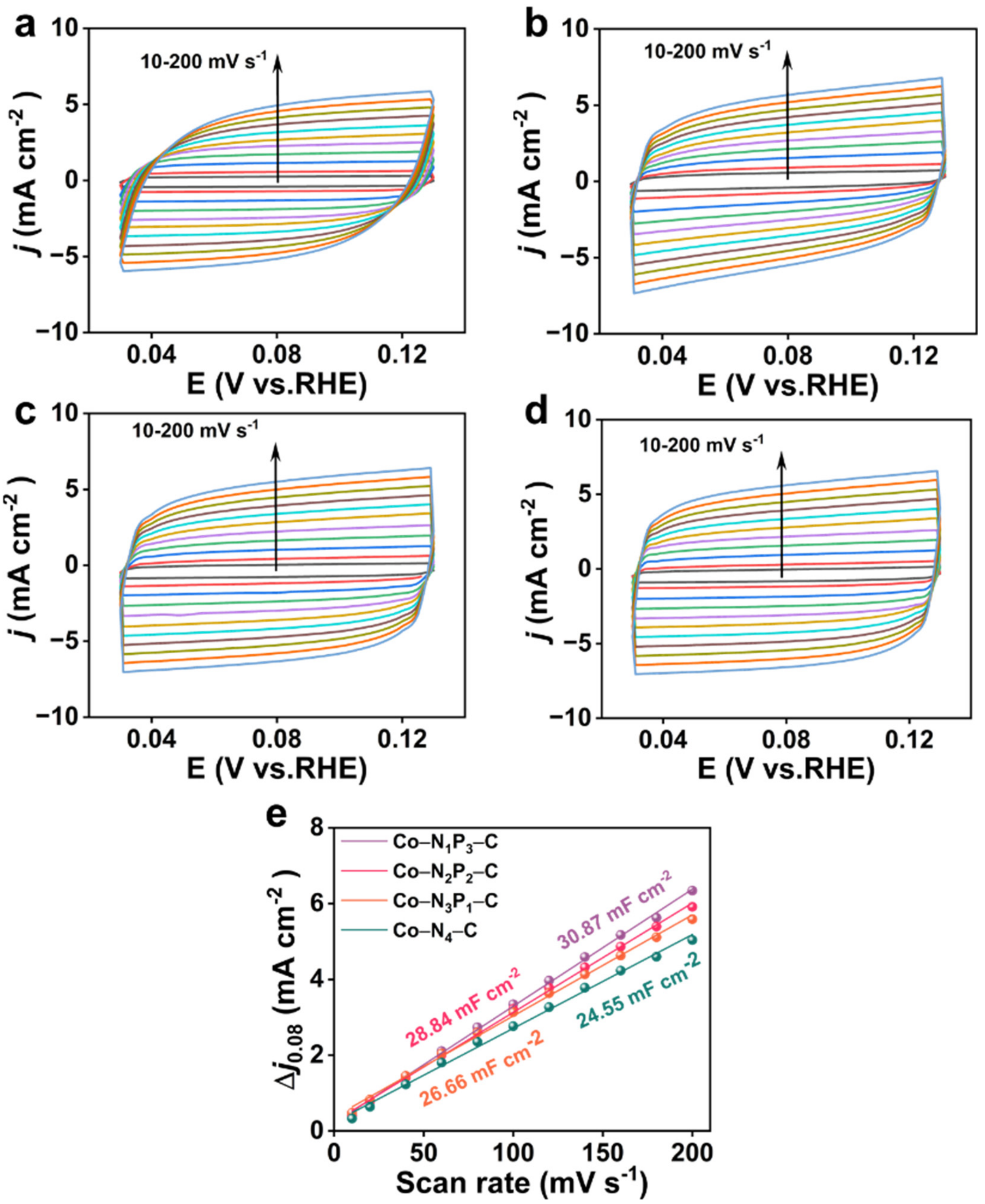

Supplementary Fig. 34: ECSA measurements. CVs for (**a**) Co–N_4–C, (**b**) Co–P_1N_3–C, (**c**) Co–P_2N_2–C, and (**d**) Co–P_3N_1–C at scan rates from 10 to 200 mV s^{-1}. (**e**) The capacitive currents at 0.08 V vs. RHE as a function of scan rate for corresponding catalysts ($\Delta j_0 = j_a - j_c$).

The calculation for Turnover frequency (TOF).

The TOF value was calculated based on estimated numbers of active centers[11]:

$$\mathrm{TOF} = \frac{\text{total hydrogen turn overs per geometric area}}{\text{surface active sites per geometric area}} \quad (3)$$

The values of the total hydrogen turnovers were calculated from the current density obtained in the HER polarization:

$$\text{HER: } \mathrm{H_2} = J_{\mathrm{HER}} * \frac{\mathrm{mA}}{\mathrm{cm^2}} * \frac{1\ \mathrm{C\ s^{-1}}}{1000\ \mathrm{mA}} + \frac{1\ \mathrm{mol\ e^{-1}}}{9645.353\ \mathrm{C}} * \frac{1\ \mathrm{mol\ H_2}}{2\ \mathrm{mol\ e^{-1}}} * \frac{6.022*16^{23}\ \mathrm{molecules}}{1\ \mathrm{mol\ H_2}} = 3.12 * 10^{15} + \frac{\mathrm{H_2 s^{-1}}}{\mathrm{cm^2}} * \mathrm{per}\frac{\mathrm{mA}}{\mathrm{cm^2}} \quad (4)$$

We assume that all Co atoms in the catalysts are active for HER. The number of Co atoms in $Co–P_2N_2–C$ was calculated from the Co molar mass and the mass loading on the carbon fiber paper. The Co content determined by ICP-OES was 4.36 wt.% and the mass loading is *ca*. 0.7 mg cm^{-2}.

$$\text{Surface active site} = \frac{4.36}{100} * \frac{0.7\ \mathrm{mg}}{\mathrm{cm^2}} * \frac{1}{58.933\ \mathrm{mg\ mol}} * \frac{6.022 * 10^{20}\ \text{sites}}{\mathrm{mol}} \quad (5)$$

$$= 3.12 * 10^{17}\ \text{sites cm}^{-2}$$

For $Co–P_2N_2–C$ catalyzed HER, it requires an overpotential of 200 mV to achieve the current density of 199.6 mA cm^{-2}, thus:

$$\mathrm{TOF(200\ \ mV)} = \frac{3.12*10^{15}*\frac{\mathrm{H_2\ \ s^{-1}}}{\mathrm{cm^2}}\ \text{per} *199.6}{3.12*10^{17}\ \text{sites per cm}^{-2}} = 2.0\ \ \mathrm{s^{-1}} \quad (6)$$

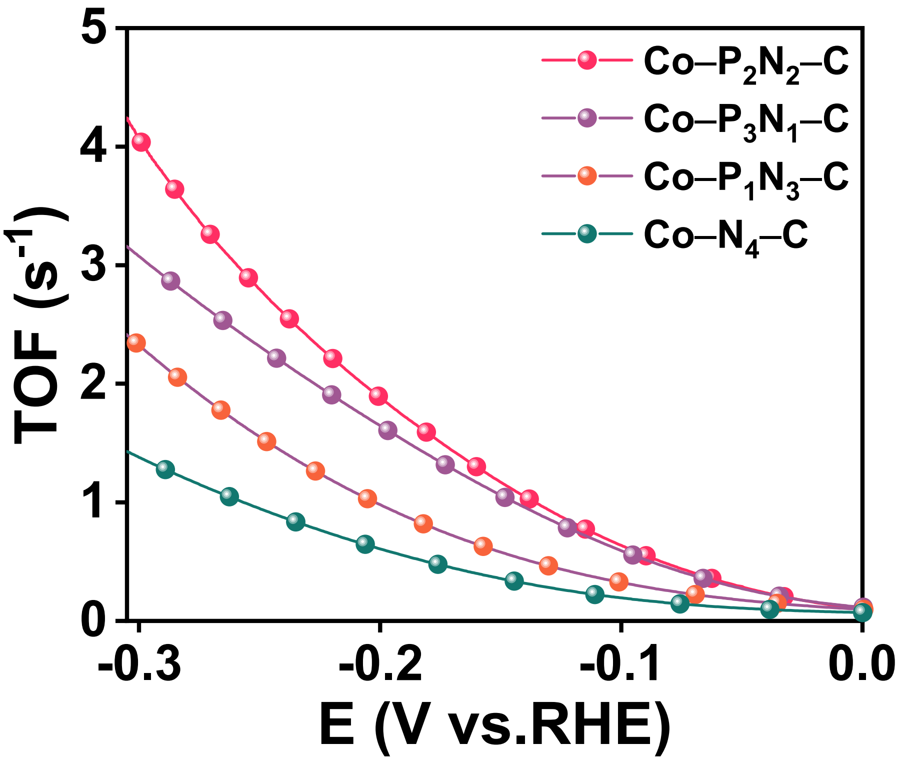

Supplementary Fig. 35: TOF calculations. The TOF values of Co–P$_2$N$_2$–C, Co–P$_3$N$_1$–C, Co–P$_1$N$_3$–C and Co–N$_4$–C in alkaline HER.

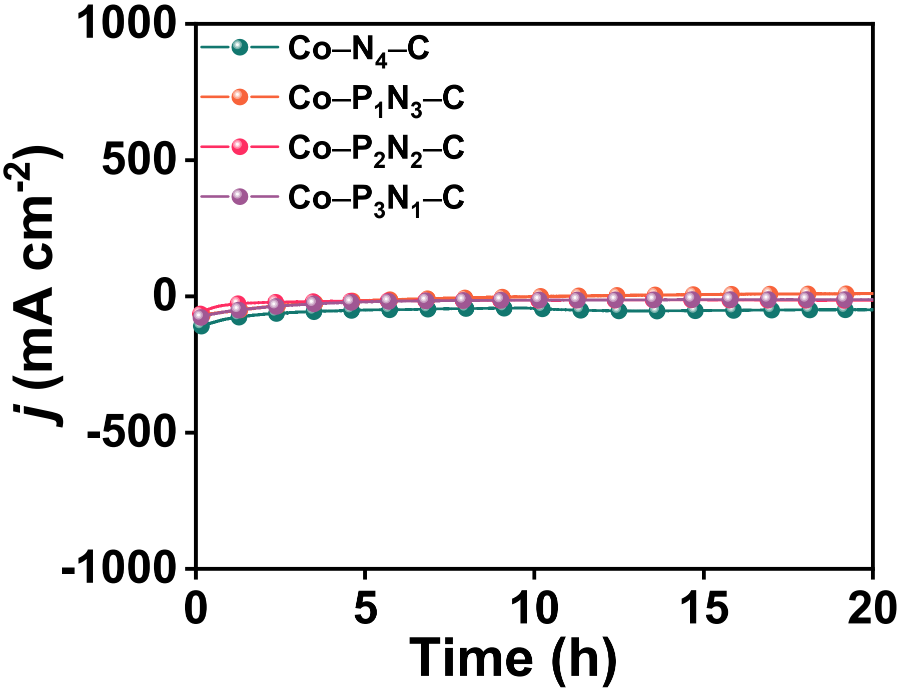

Supplementary Fig. 36: Stability test. The long-term stability measurement of HER for $Co–N_4–C$, $Co–P_1N_3–C$, $Co–P_2N_2–C$ and $Co–P_3N_1–C$.

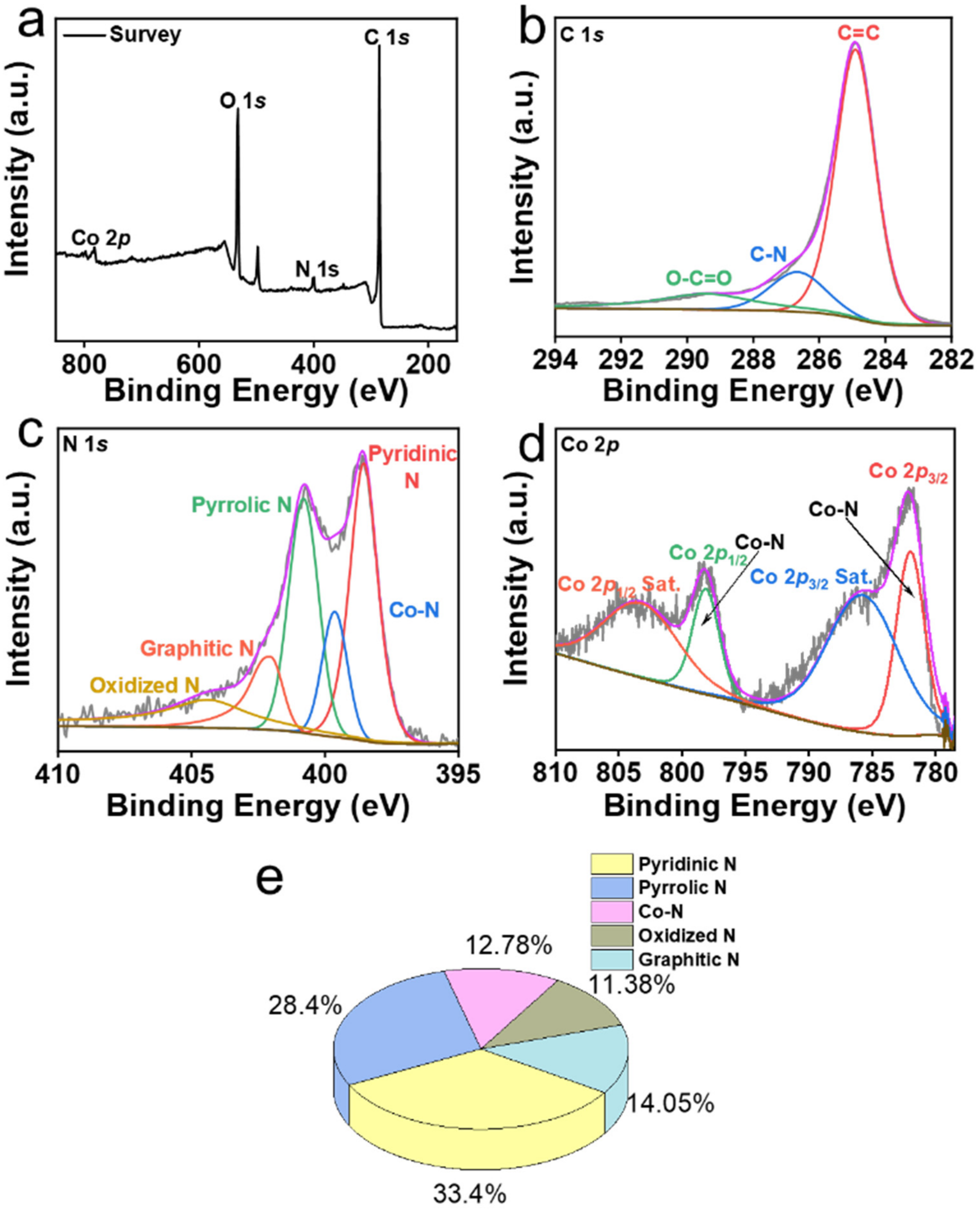

Supplementary Fig. 37: XPS analysis of non-helix Co–N4–C. (**a**) XPS survey spectrum, (**b**) C 1*s*, (**c**) N 1*s*, (**d**) Co 2*p* spectra of non-helix Co–N4–C. (**e**) Distribution of N element with different bonding configurations.

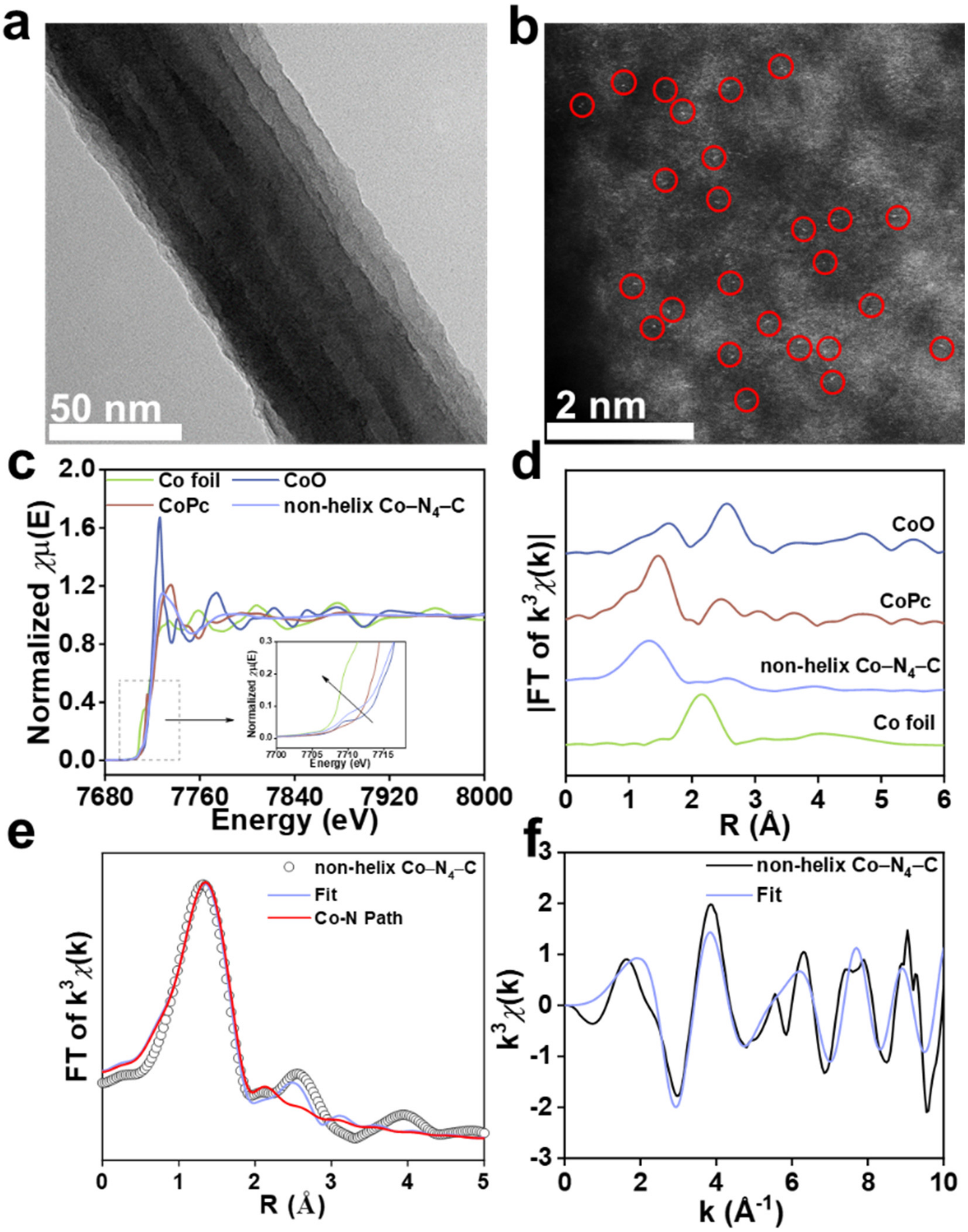

Supplementary Fig. 38: Characterizations of the non-helix Co–N$_4$–C. (**a**) TEM and (**b**) the high-resolution HAADF-STEM image with spherical aberration correction for non-helix Co–N$_4$–C. (**c**) Co K-edge XANES spectra of non-helix Co–N$_4$–C, and reference Co foil, CoO, CoPc. (**d**) Corresponding Fourier-transformed (FT) k^3-weighted EXAFS spectra. (e) The corresponding k^3-weighted EXAFS fitting curves at k space for the non-helix Co–N$_4$–C. (f) FT-EXAFS fitting curves of non-helix Co–N$_4$–C at k space.

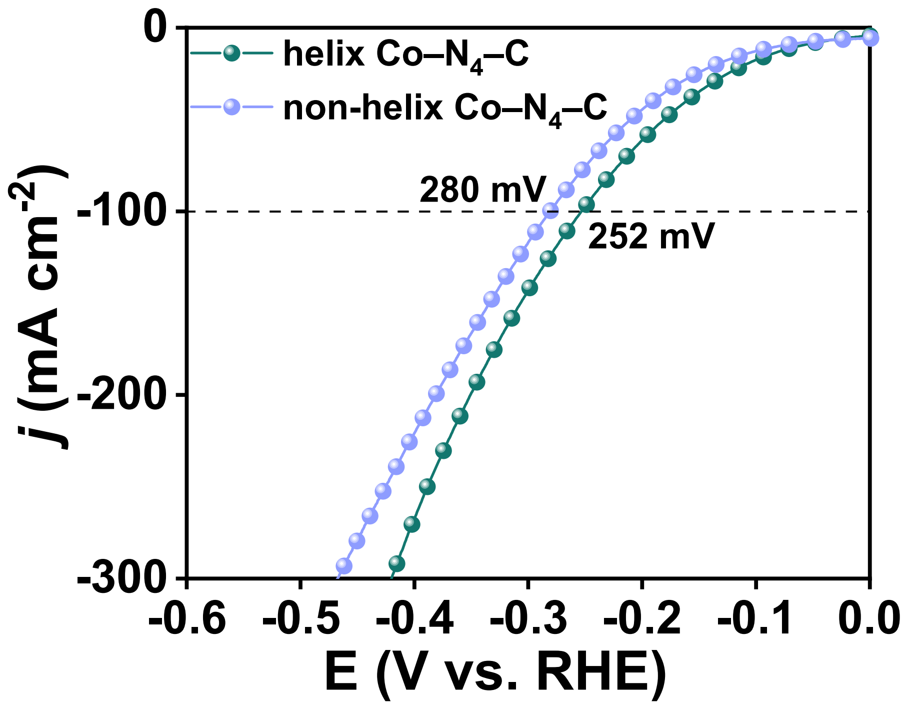

Supplementary Fig. 39: HER performance of non-helix and helix Co–N$_4$–C. Polarization curves of non-helix and helix Co–N$_4$–C in 1.0 M KOH electrolyte. The scan rate was 5 mV s^{-1}.

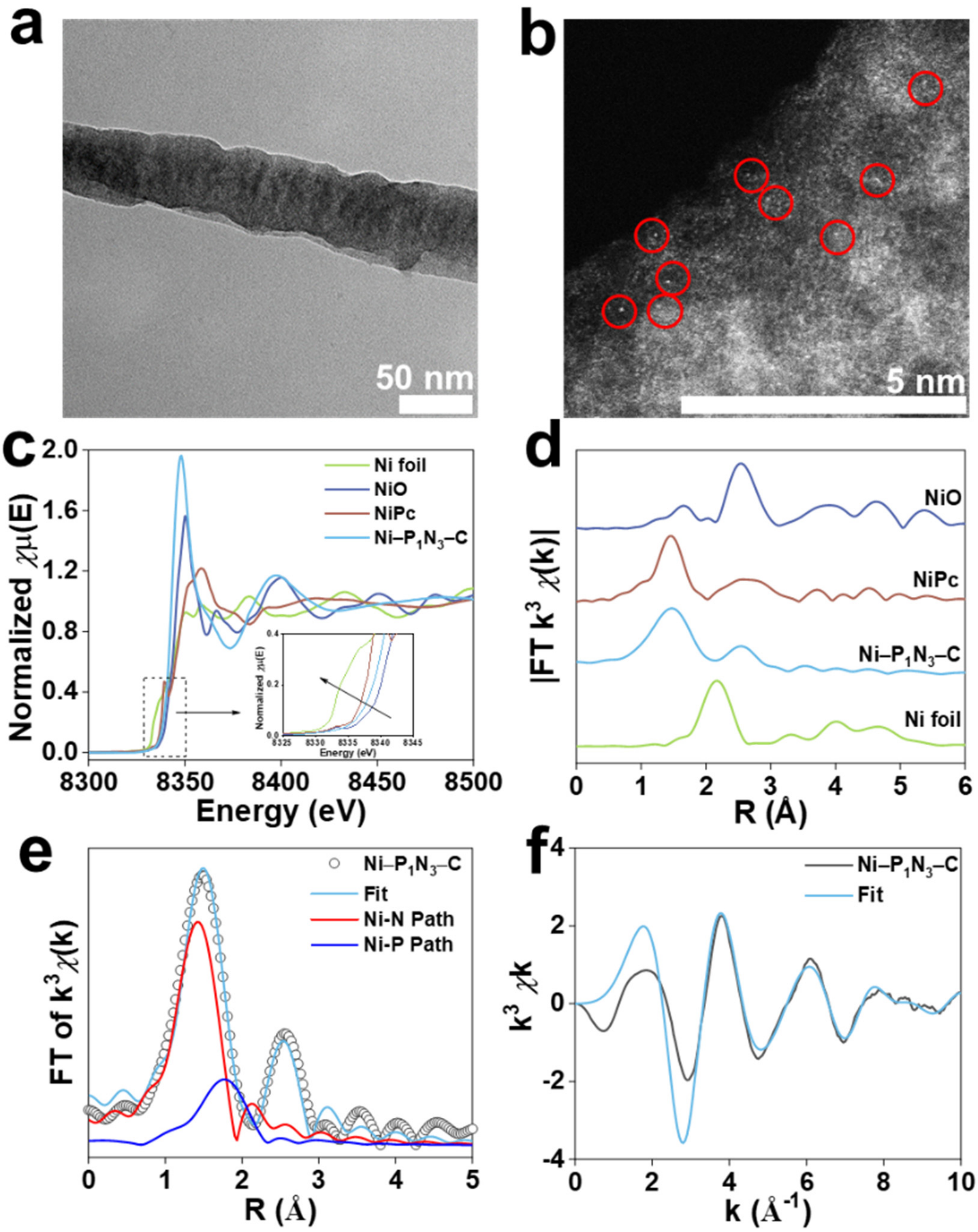

Supplementary Fig. 40: Characterization of Ni–P_1N_3–C catalyst. (**a**) TEM, (**b**) HAADF-STEM image. (**c**) XAFS curves. (**c**) Ni K-edge XANES spectra of Ni–P_1N_3–C, and reference Ni foil, NiO, NiPc. (**d**) Fourier-transformed (FT) k^3-weighted EXAFS spectra of Ni–P_1N_3–C, and reference Ni foil, NiO, NiPc. (**e**) The k^3-weighted EXAFS fitting curves at R space for Ni–P_1N_3–C. (**f**) FT-EXAFS fitting curves of Ni–P_1N_3–C at k space.

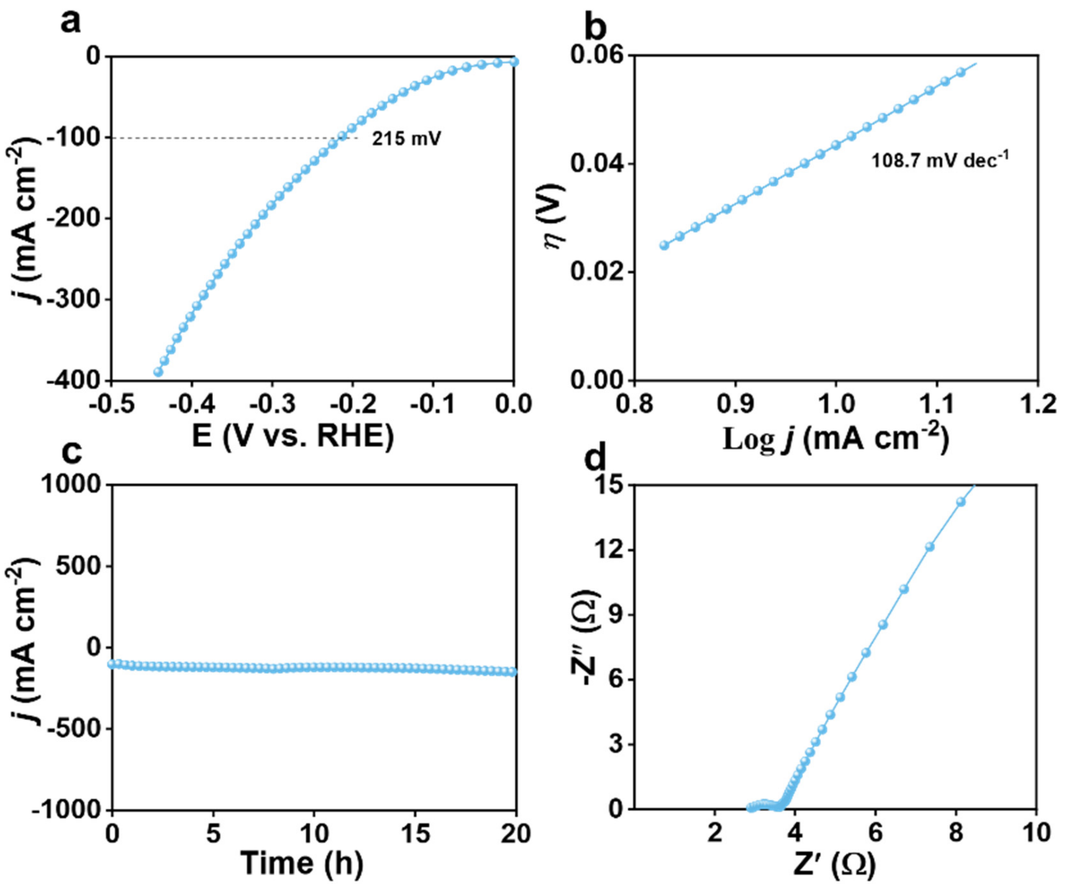

Supplementary Fig. 41: HER performance of Ni–P$_1$N$_3$–C: (a) Polarization curve, (**b**), Tafel plot, (**c**), Nyquist plot, and (**d**) Stability test of Ni–P$_1$N$_3$–C in 1.0 M KOH electrolyte. The scan rate was 5 mV s^{-1}.

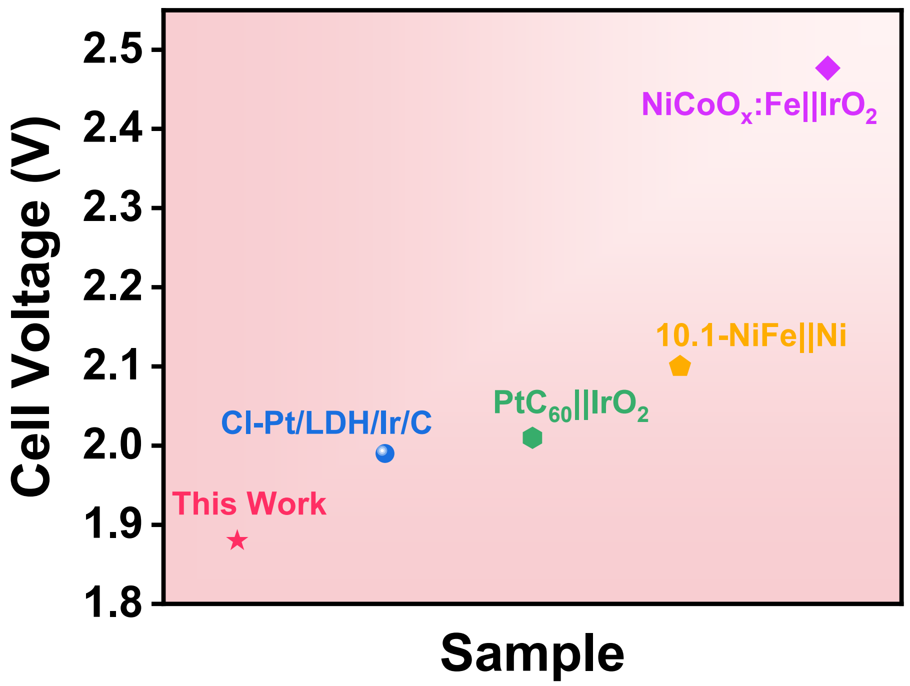

Supplementary Fig. 42: Electrocatalysis performance comparison. Comparison of the cell voltages at 1.0 A cm^2 for NiFe-MOF@GQD || Co–P_2N_2–C couple with other advanced electrolysers[12-15].

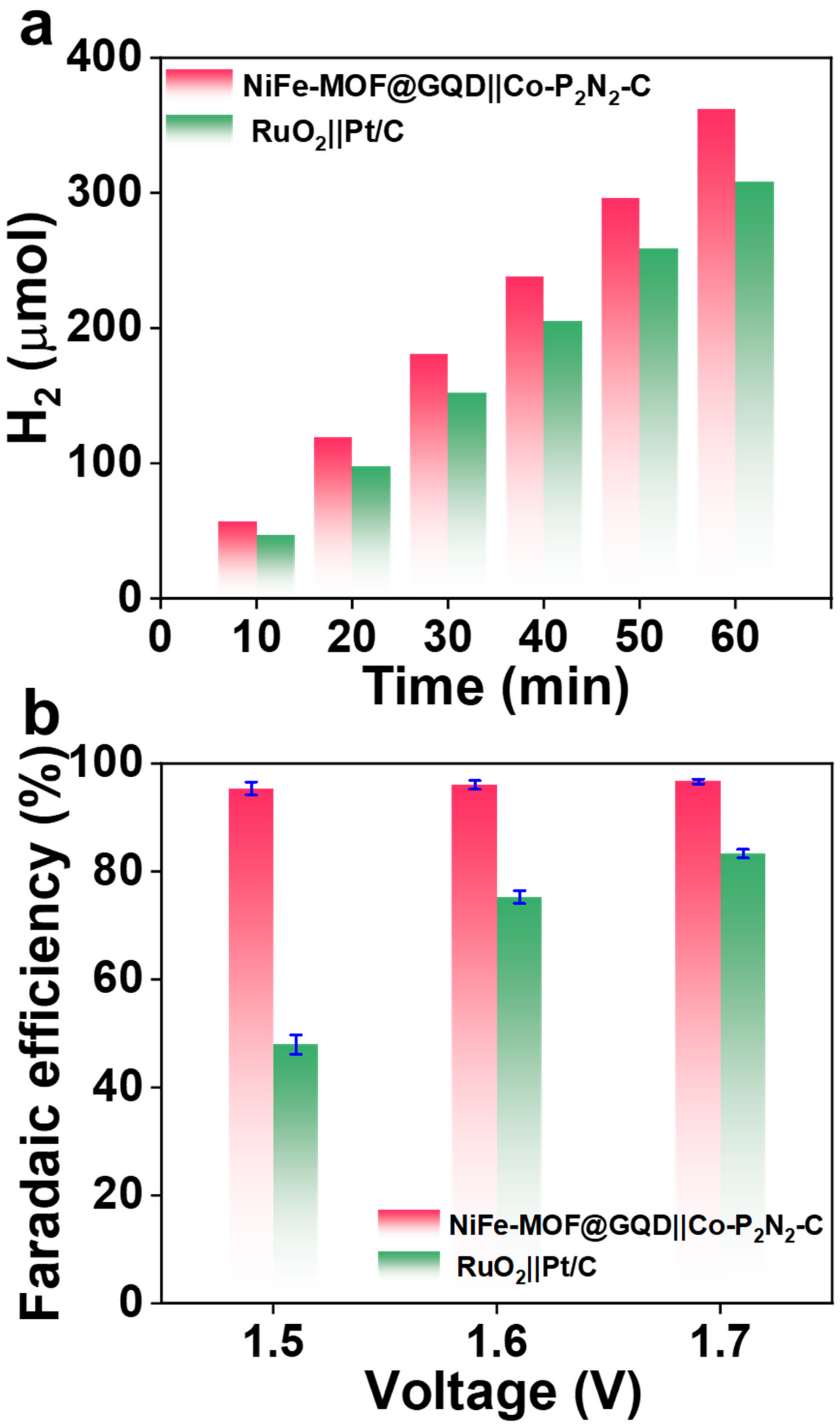

Supplementary Fig. 43: The electrocatalytic HER efficiency of Co–P_2N_2–C. (**a**) Hydrogen production at specific current of 100 mA for Co–P_2N_2–C and Pt/C. (**b**) Faradaic efficiency at a specific voltage (1.5, 1.6, and 1.7 V) of Co–P_2N_2–C and Pt/C-based electrolysers. The error bar reflects three measurements.

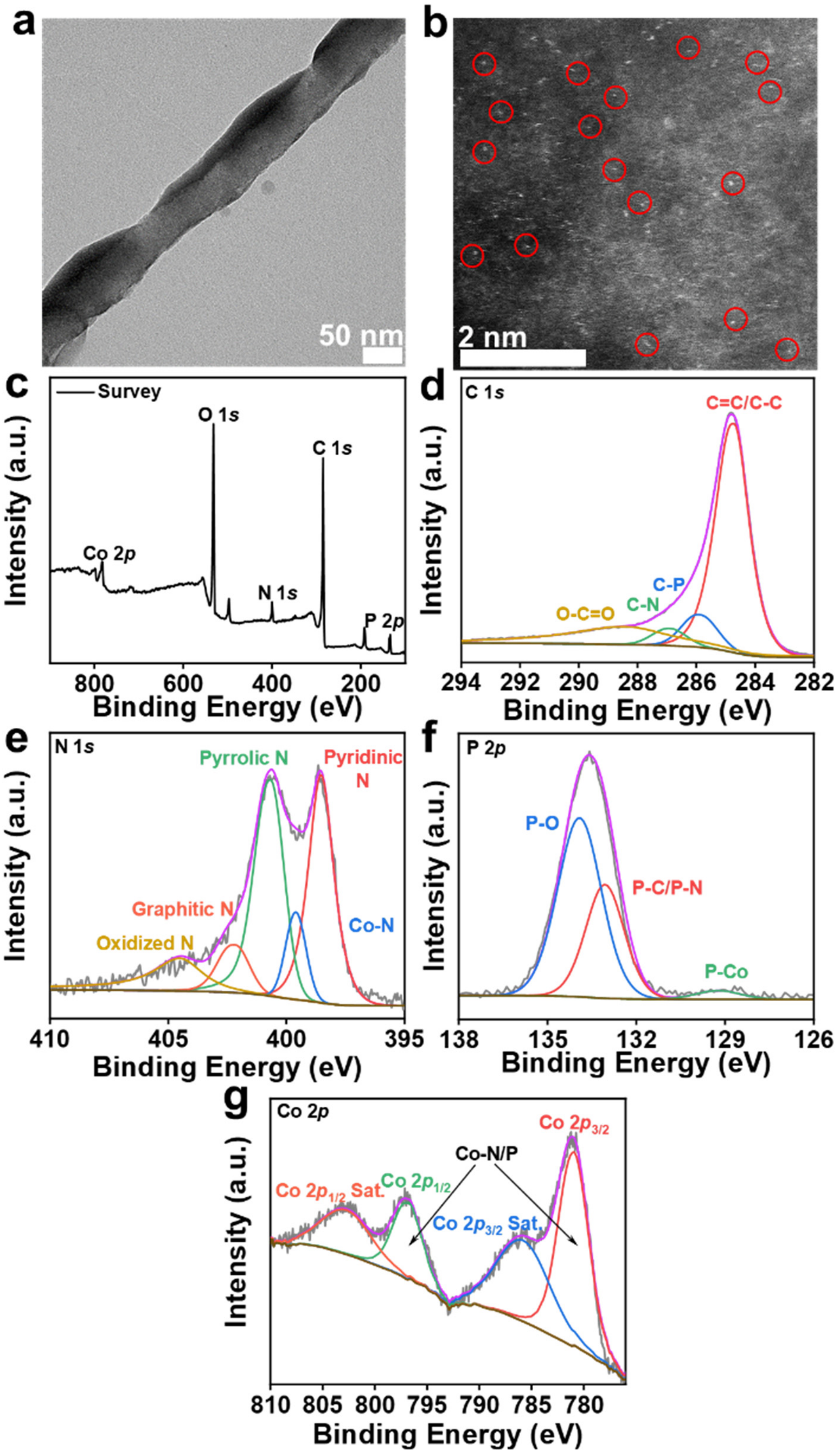

Supplementary Fig. 44: Structural characterizations and XPS analysis of the post-HER Co–P_2N_2–C catalyst. (a) TEM, (b) HAADF-STEM image (c) XPS survey spectrum, (d) C 1*s*, (e) N 1*s*, (f) P 2*p*, (g) Co 2*p* spectra.

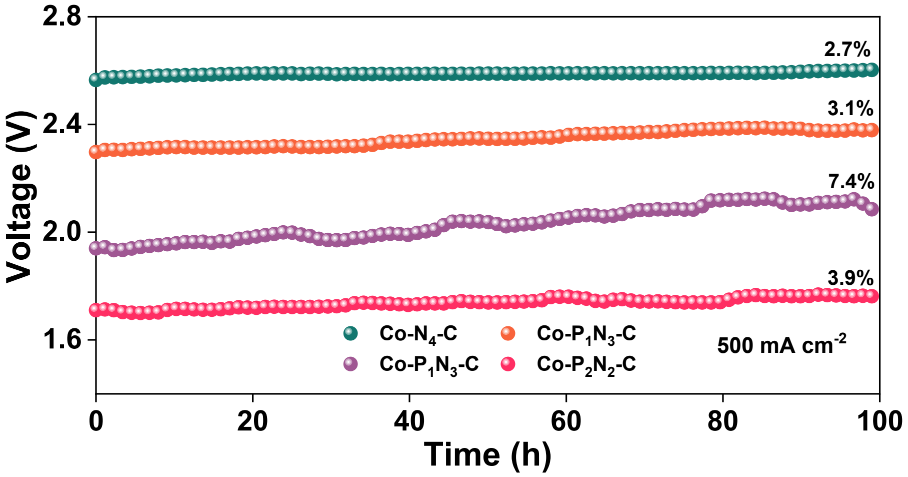

Supplementary Fig. 45: Durability test of the Co–P_xN_{4-x}–C catalysts. Chronopotentiometry curves of the Co–N_4–C, Co–P_1N_3–C, Co–P_2N_2–C, and Co–P_3N_1–C based AEM electrolyser at current density of 500 mA cm^{-2}.

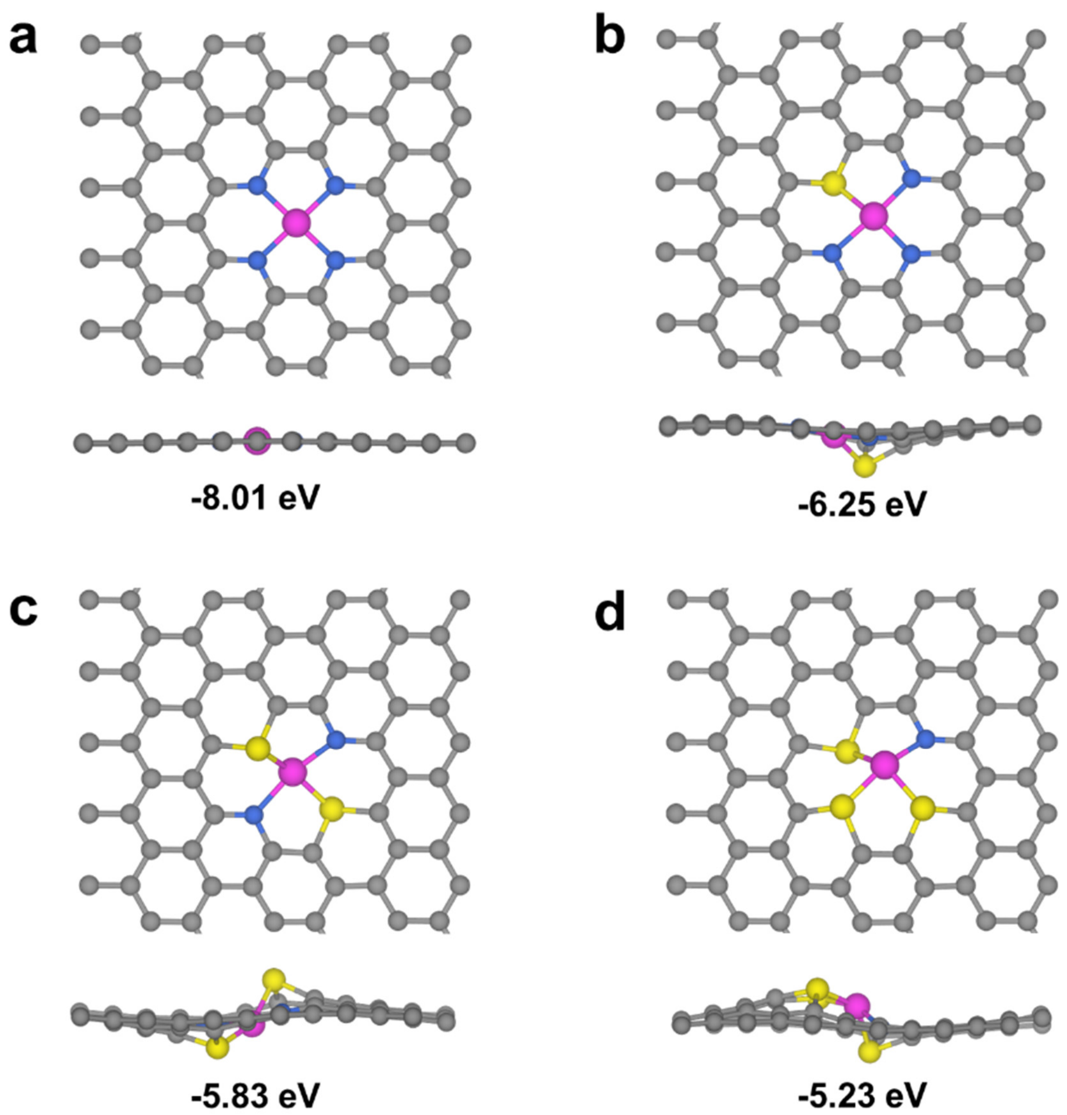

Supplementary Fig. 46: Theoretical models. Theoretical model of (**a**) Co–N_4–C, (**b**) Co–P_1N_3–C, (**c**) Co–P_2N_2–C, and (**d**) Co–P_3N_1–C with corresponding energies. The gray, red, blue and yellow balls represent for C, Co, N, and P atoms, respectively.

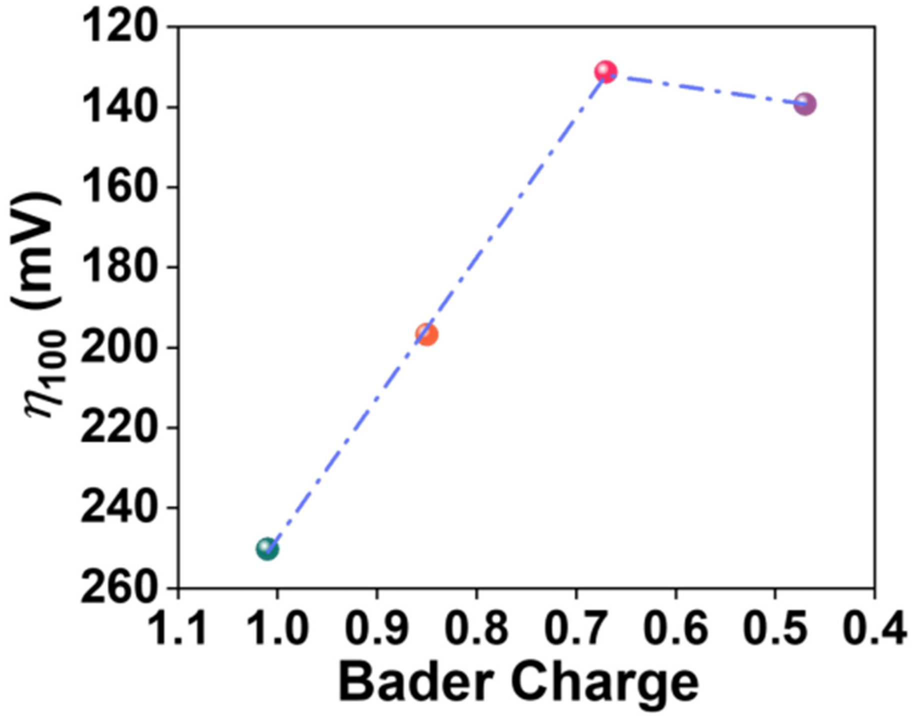

Supplementary Fig. 47: The volcanoes of HER overpotential for the Bader charge of active center Co atom.

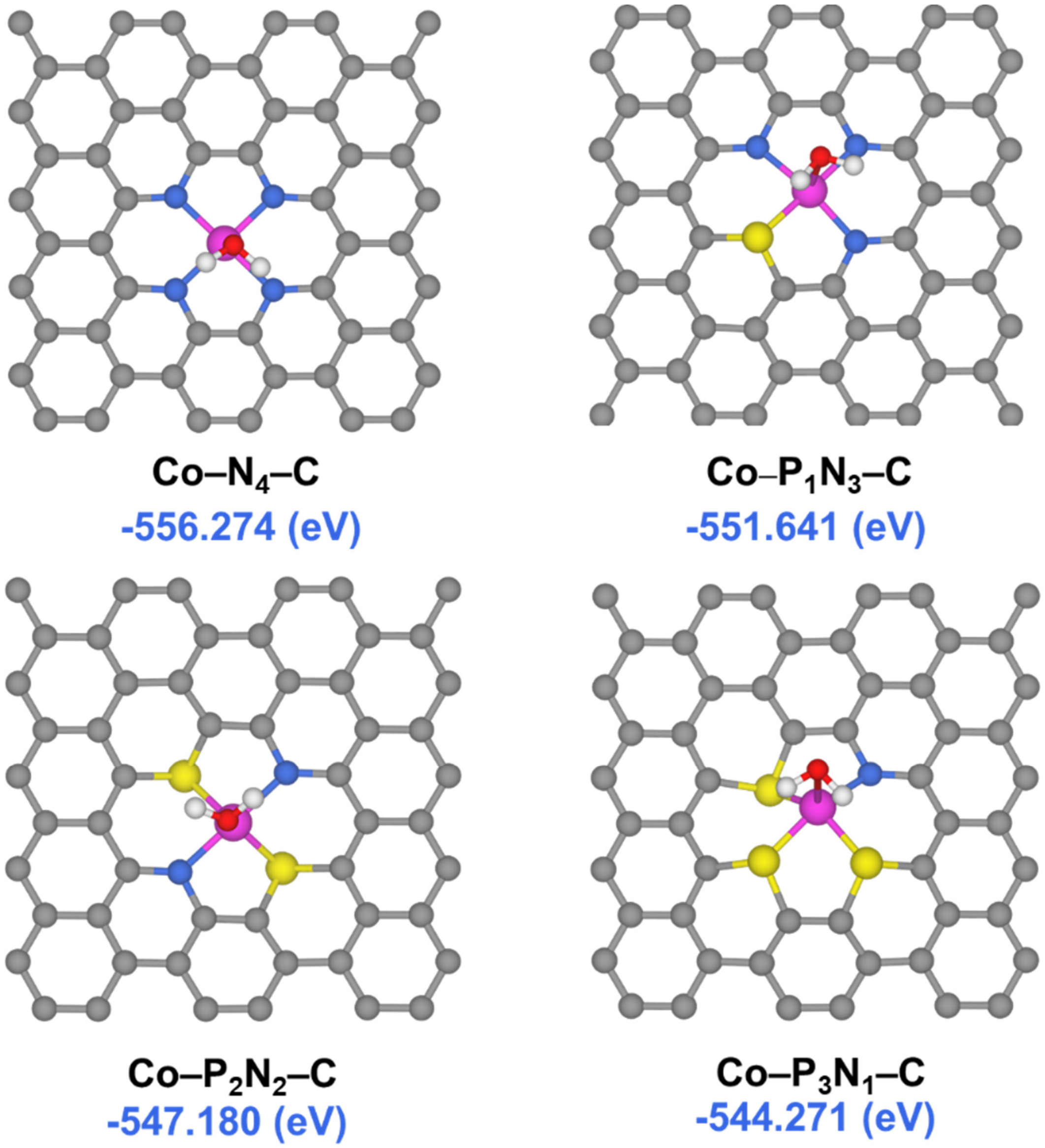

Supplementary Fig. 48: Theoretical models of adsorbed H_2O on Co–P_xN_{4-x}–C catalysts. The intermediate structures of H_2O^* on the model of Co–P_xN_{4-x}–C catalysts with corresponding total energies. The gray, red, blue and yellow balls represent for C, Co, N, and P atoms, respectively.

The adsorption energy of H_2O is calculated according to the equation: $E_{ads} = E_{total} - E_{catalyst} - E_{H_2O}$, where E_{total} represents the total energy, $E_{catalyst}$ and E_{H_2O} are the energy of the catalyst model and the energy of adsorbed H_2O, respectively.

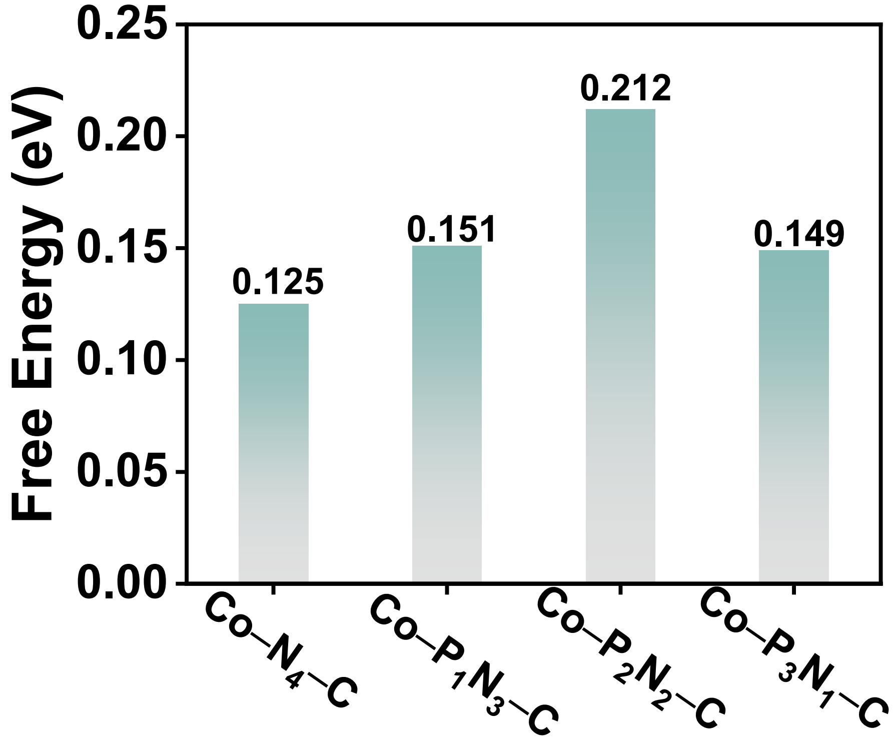

Supplementary Fig. 49: H_2O adsorption free energy calculations. The free energy of H_2O adsorption at Co sites in Co–N_4–C, Co–P_1N_3–C, Co–P_2N_2–C and Co–P_3N_1–C.

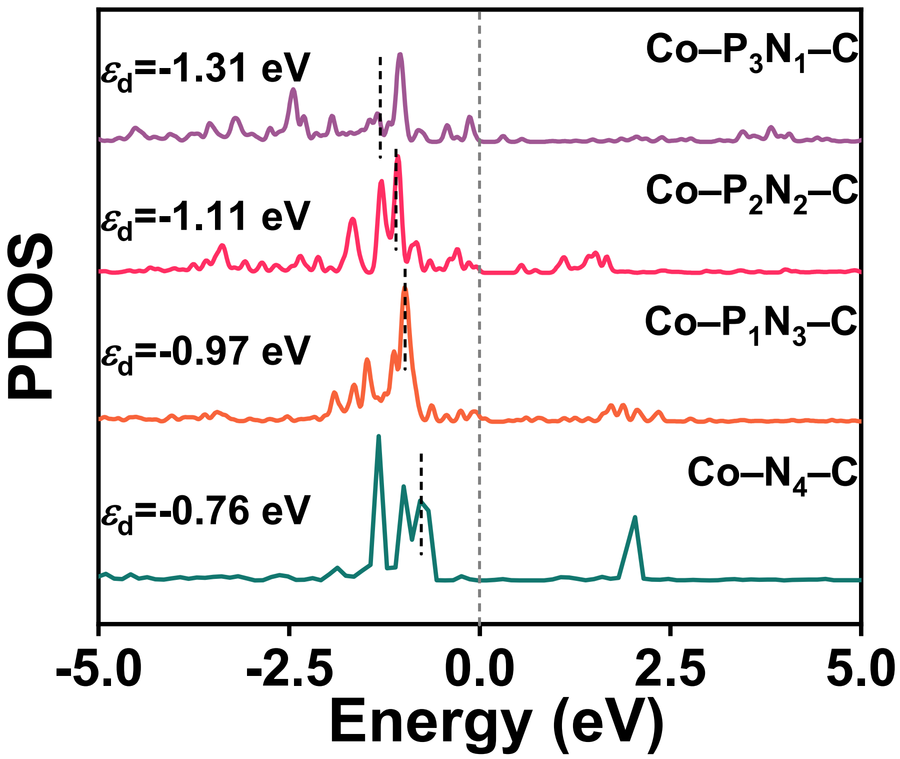

Supplementary Fig. 50: Projected density of states (PDOS). Projected density of states (PDOS) for *d* orbital of Co single atom in various Co–P_xN_{4-x}–C (x=0, 1, 2, 3).

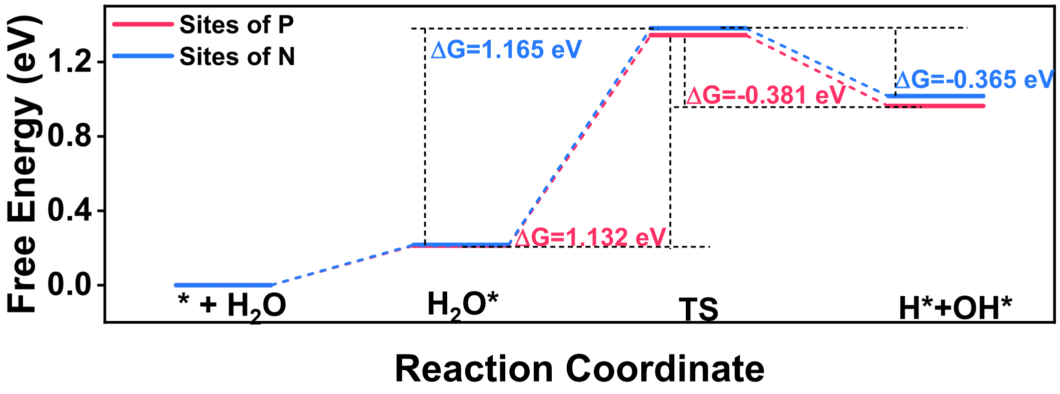

Supplementary Fig. 51: Free energy calculation. Bonding thermodynamics priority of the reactants adsorption on N and P sites in Co–P_2N_2–C.

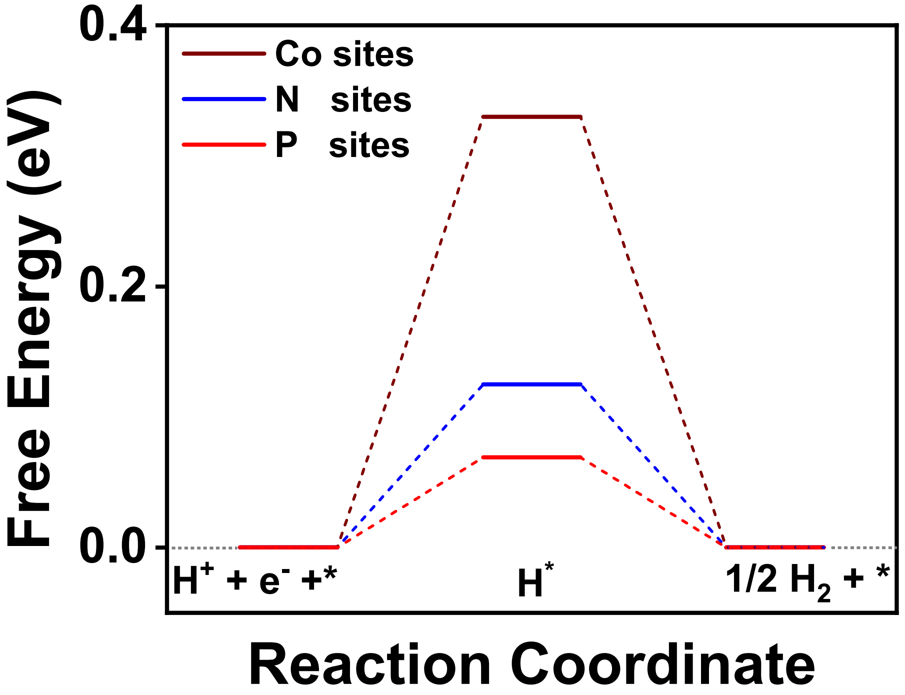

Supplementary Fig. 52: H adsorption energy calculation. The △G_{H*} at the Co site compared with P and N sites in Co–P_2N_2–C.

The inferior △G_{H*} of Co sites in Co–P_2N_2–C suggests the energetically unfavorable adsorption of H* compared with that of P sites (0.069 eV vs. 0.330 eV).

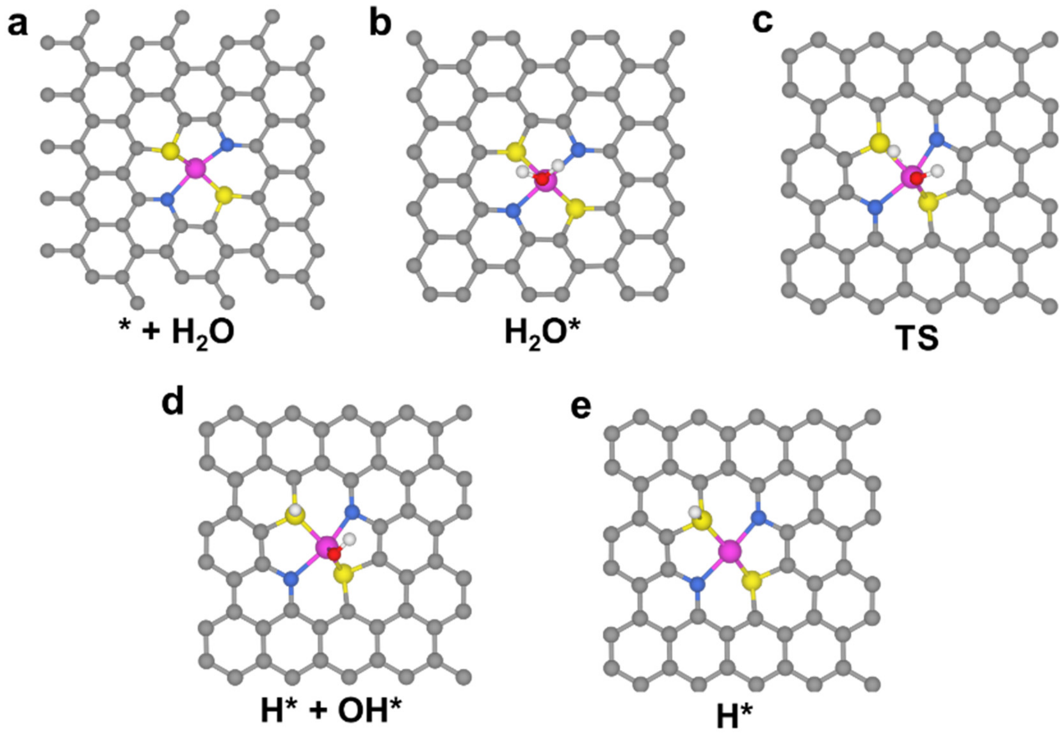

Supplementary Fig. 53: The configurations of Volmer-Heyrovsky mechanism for alkaline HER on Co–P_2N_2–C. The adsorption and dissociation of H_2O molecule on Co site (**a-c**) and H transfer to P site (**d, e**) during the HER.

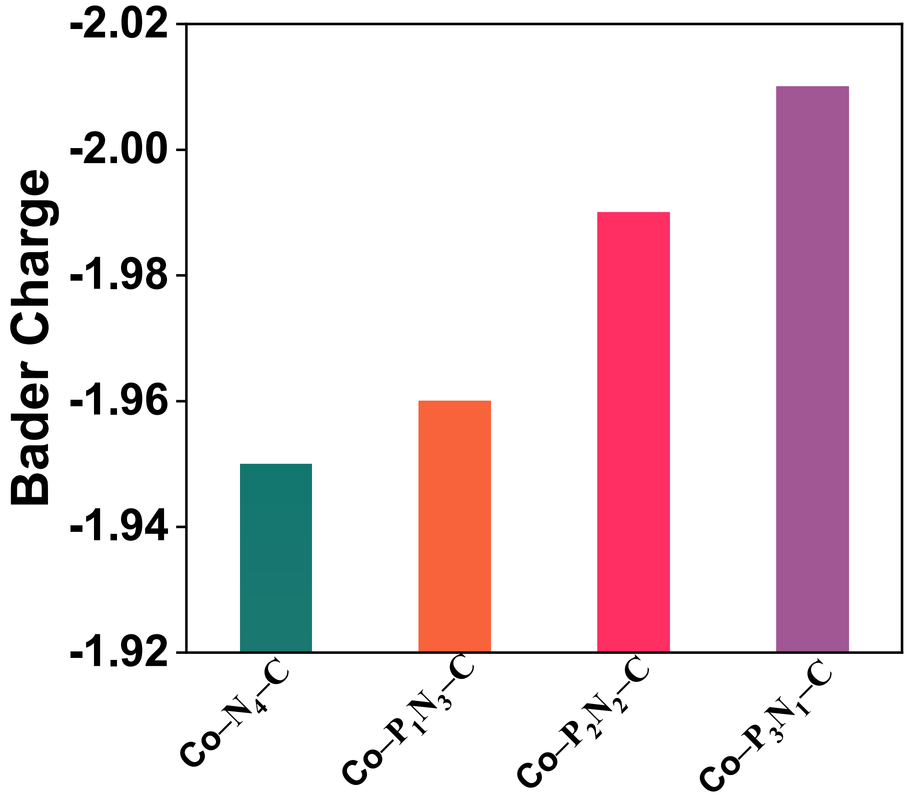

Supplementary Fig. 54: Bader charge analysis. The calculated Bader charge of adsorbed H_2O over Co–P_xN_{4-x}–C.

Supplementary Tables

Supplementary Table 1: Atomic composition analysis. Summary of the atomic content in NGQDs with different A/H.

Samples	C 1s (at.%)	N 1s (at.%)	O 1s (at.%)	A/H
NGQDs	70.83	10.93	18.24	0.69
NGQDs-1	70.73	10.44	18.81	0.66
NGQDs-2	70.81	10.12	19.07	0.61
NGQDs-3	70.58	9.62	19.8	0.54
NGQDs-4	70.72	7.89	21.39	0.43

Supplementary Table 2: Elemental analysis for Co–PN–C catalysts. Elemental contents of Co, C, N, P, and O based on ICP-OES (wt%) and XPS (at%) for Co–PN–C catalysts.

Samples	C (at%)	N (at%)	O (at%)	P (at%)	Co (at%)/(wt%)
Co–N_4–C	72.06	6.72	18.9	#	2.32/4.65
Co–P_1N_3–C	70.6	5.92	18.1	3.14	2.24/4.43
Co–P_2N_2–C	68.83	4.98	19.21	4.71	2.27/4.41
Co–P_3N_1–C	71.38	3.94	17.4	4.92	2.36/4.57

Supplementary Table 3: EXAFS fitting of the Co-PN-C catalysts. EXAFS fitting parameters at the Co K-edge various samples (S_0^2=0.95).

samples	path	*C. N.*[a]	*R* (Å)[b]	σ^2 (× 10^{-3} Å^2)[c]	Δ*E* (eV)[d]	*R* factor[e]
Co–N_4–C	Co-N	3.9±0.8	1.93±0.02	3.8±3.3	-7.3±2.4	0.01
Co–P_1N_3–C	Co-N	3.1±1.7	1.90±0.03	9.9±5.1	-4.0±3.8	0.01
	Co-P	0.9±0.6	2.28±0.03	4.7±1.9		
Co–P_2N_2–C	Co-N	2.1 ± 0.2	1.92±0.03	9.8±3.3	-6.7±2.7	0.02
	Co-P	1.9 ± 0.3	2.30±0.03	4.6±3.2		
Co–P_3N_1–C	Co-N	0.9±0.3	1.92±0.02	9.5±0.9	-4.3±6.2	0.02
	Co-P	2.9±1.1	2.28±0.03	6.6±5.4		
Non-helix Co–N_4–C	Co-N	5.8±0.8	2.10±0.01	8.5±2.4	0.1±1.1	0.02
Co foil	Co-Co	12*	2.51*	6.2±0.1	7.1±0.2	0.01
CoPc	Co-N	4*	1.93*	2.3±2.2	5.7±2.2	0.02
	Co-N-C	8*	2.96*	4.1±1.4		
CoO	Co-O	6*	2.13*	5.2±2.9	-3.0±1.0	0.01
	Co-O-Co	12*	3.01*	11.4±1.5		
Co_2O_3	Co-O	3*	1.96*	2.8±0.1	-1.6±8.6	0.01
	Co-O	3*	2.27*	9.6±1.6		
	Co-O-Co	5*	3.06*	1.9±1.6		
	Co-O-Co	2*	3.26*	12.5±4.9		

[a]*C. N.*: coordination numbers; [b]*R*: bond distance; [c]σ^2: Debye-Waller factors; [d]ΔE_0: the inner potential correction. [e]*R* factor: goodness of fit. *The experimental EXAFS fit by fixing *C. N.* as the known crystallographic value.

Supplementary Table 4: Summary of HER performances. The comparison of alkaline HER performance of advanced single atom catalysts.

Electrocatalysts	η_{10} (mV)	Tafel slope (mV dec^{-1})	Ref.
Pt@PCM	139	73.6	[16]
A–CoPt–NC	50	48	[17]
$Mo_1N_1C_2$	132	86	[18]
Fe-N_4SAs/NPC	202	123	[19]
Co_1/PCN	89	52	[20]
Ir_1@Co/NC	55	119	[21]
Ru@Co–SAs/N–C	7	30	[22]
Ni_5P_4–Ru	54	52	[23]
SANi–PtNWs	70	60.3	[24]
NiSA–MoS_2/CC	98	75	[25]
Ni–MoS_2	98	60	[26]
Pt/np–$Co_{0.85}$Se	58	39	[27]
Ru/ECM	83	59	[28]
Co–P_2N_2–C	38	66.9	This work

Supplementary Table 5: **EXAFS fitting of the Ni–P_1N_3–C catalyst.** EXAFS fitting parameters at the Ni K-edge various samples (S_0^2=0.86).

sample	path	C. N.[a]	R (Å) [b]	σ^2 (× 10^{-3} $Å^2$) [c]	ΔE (eV) [d]	R factor[e]
Ni–P_1N_3–C	Ni-N	3.3±0.5	2.00±0.02	9.5±6.7	-2.5±5.7	0.02
	Ni-P	0.9±0.2	2.31±0.02	7.5±3.6		

[a]C. N.: coordination numbers; [b]R: bond distance; [c]σ^2: Debye-Waller factors; [d]ΔE_0: the inner potential correction. [e]R factor: goodness of fit.

Supplementary Table 6: Composition analysis of the post-HER Co–P_2N_2–C catalyst. ICP test of the post-HER Co–P_2N_2–C.

Samples	Co (wt%)
Co–P_2N_2–C	4.43
Co–P_2N_2–C (post-HER)	4.37

Supplementary Table 7: Theoretical calculations for the formation energy, binding energy, and cohesive energy of all Co–P_xN_{4-x}. The comparison of $E_{formation}$, $E_{binding}$ - $E_{cohesive}$ values for all Co–P_xN_{4-x} models.

Samples	$E_{formation}$ (eV)	$E_{binding}$-$E_{cohesive}$ (eV)	Models
Co-N_4-C	-8.01	-2.65	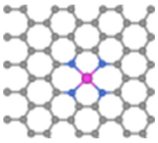
Co-P_1N_3-C	-6.25	-0.99	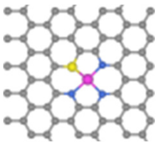
Co-P_2N_2-C (para)	-5.83	-0.47	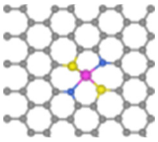
Co-P_2N_2-C (ortho)	-4.02	1.34	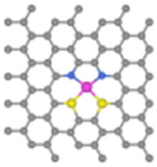
Co-P_3N_1-C	-5.23	0.13	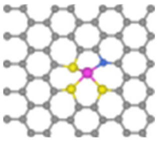

Supplementary Table 8: The bond length changes during MD simulations. The static, peak and difference of bond length for Co–N and Co–P in Co–P_xN_{4-x}–C catalysts during MD simulations.

Samples	N/P position	Static bond length (Å)	Peak bond length (Å)	Difference (Å)
Co-N_4-C	Co-N	1.8675	1.8675	0
Co-P_1N_3-C	Co-N1	1.8861	1.9425	0.06
	Co-N2	1.8634	1.8525	-0.01
	Co-N3	1.9358	1.8525	-0.08
	Co-P	2.0742	2.1075	0.03
Co-P_2N_2-C	Co-N1	1.8944	1.889	-0.005
	Co-N2	1.9136	1.9174	-0.003
	Co-P1	2.0292	2.1132	0.08
	Co-P2	2.1418	2.1994	0.06
Co-P_3N_1-C	Co-N	1.8529	1.8615	0.01
	Co-P1	2.0407	2.0825	0.04
	Co-P2	2.0692	2.2015	0.13
	Co-P3	2.1906	2.1165	-0.07

Supplementary References:

1. Wang, L. et al. Gram-scale synthesis of single-crystalline graphene quantum dots with superior optical properties. *Nat. Commun.* **5**, 5357 (2014).

2. Hess, B., Kutzner, C., Van Der Spoel, D. & Lindahl, E. GROMACS 4: algorithms for highly efficient, load-balanced, and scalable molecular simulation. *J. Chem. Theory Comp*. **43**, 435–447 (2008).

3. Chen, L. et al. Organic–inorganic cobalt-phosphonate-derived hollow cobalt phosphate spherical hybrids for highly efficient oxygen evolution. *ACS Sustain. Chem. Eng.* **7**, 13559–13568 (2019).

4. Hu, B. et al. Atomic Co/Ni dual sites with N/P-coordination as bifunctional oxygen electrocatalyst for rechargeable Zinc-air batteries. *Nano Res.* **14**, 3482–3488 (2021).

5. Wei, X. et al. Cross-linked polyphosphazene hollow nanosphere-derived N/P-doped porous carbon with single nonprecious metal atoms for the oxygen reduction reaction. *Angew. Chem. Int. Ed.* **132**, 14747–14754 (2020).

6. Li, J. et al. Boosted ammonium production by single cobalt atom catalysts with high faradic efficiencies. *Proc. Natl. Acad. Sci.* **119**, e2123450119 (2022).

7. Matsumoto, I., Sekiya, R. & Haino, T. Self-assembly of nanographenes. *Angew. Chem. Int. Ed*. **60**, 12706–12711 (2021).

8. Grimme, S. Semiempirical GGA-type density functional constructed with a long-range dispersion correction. *J. Comput. Chem*. **27**, 1787-1799 (2006).

9. Sun, D. et al. In-situ phosphating Co@ Nitrogen-doping graphene boosts overall water splitting under alkaline condition. *J. Electroanal. Chem*. **904**, 115882 (2022).

10. Henkelman, G., Arnaldsson, A. & Jónsson, H. A fast and robust algorithm for Bader decomposition of charge density. *Comput. Mater. Sci*. **36**, 354-360 (2006).

11. Mathew, K., Sundararaman, R., Letchworth-Weaver, K., Arias, T. A. & Hennig, R. G. Implicit solvation model for density-functional study of nanocrystal surfaces and reaction pathways. *J. Chem. Phys*. **140**, 084106 (2014).

12. Zhang, T. et al. Pinpointing the axial ligand effect on platinum single-atom-catalyst towards efficient alkaline hydrogen evolution reaction. *Nat. Commun*. **13**, 6875 (2022).

13. Chen, J. et al. Diversity of platinum-sites at platinum/fullerene interface accelerates alkaline hydrogen evolution. *Nat. Commun*. **14**, 1711 (2023).

14. López-Fernández, E. et al. Ionomer-Free Nickel-Iron bimetallic electrodes for efficient anion exchange membrane water electrolysis. *Chem. Eng. J.* **433**, 133774 (2022).

15. Xu, D. et al. Earth-abundant oxygen electrocatalysts for alkaline anion-exchange-membrane water electrolysis: effects of catalyst conductivity and comparison with performance in three-electrode cells. *ACS Catal.* **9**, 7-15 (2018).

16. Zhang, H. et al. Dynamic traction of lattice-confined platinum atoms into mesoporous carbon matrix for hydrogen evolution reaction. *Sci. Adv.* **4**, eaao6657 (2018).

17. Zhang, L. et al. Charge polarization from atomic metals on adjacent graphitic layers for enhancing the hydrogen evolution reaction. *Angew. Chem. Int. Ed.* **131**, 9504-9508 (2019).

18. Chen, W. et al. Rational design of single molybdenum atoms anchored on N-doped carbon for effective hydrogen evolution reaction. *Angew. Chem. Int. Ed.* **129**, 16302-16306 (2017).

19. Pan, Y. et al. A bimetallic Zn/Fe polyphthalocyanine-derived single-atom Fe-N_4 catalytic site: a superior trifunctional catalyst for overall water splitting and Zn-air batteries. *Angew. Chem. Int. Ed.* **57**, 8614-8618 (2018).

20. Cao, L. et al. Identification of single-atom active sites in carbon-based cobalt catalysts during electrocatalytic hydrogen evolution. *Nat. Catal.* **2**, 134-141 (2019).

21. Lai, W. et al. General π-electron-assisted strategy for Ir, Pt, Ru, Pd, Fe, Ni single-atom electrocatalysts with bifunctional active sites for highly efficient water splitting. *Angew. Chem. Int. Ed.* **58**, 11868-11873 (2019).

22. Yuan, S. et al. A universal synthesis strategy for single atom dispersed cobalt/metal clusters heterostructure boosting hydrogen evolution catalysis at all pH values. *Nano Energy*. **59**, 472-480 (2019).

23. He, Q. et al. Achieving efficient alkaline hydrogen evolution reaction over a Ni_5P_4 catalyst incorporating single-atomic Ru sites. *Adv. Mater.* **32**, 1906972 (2020).

24. Li, M. et al. Single-atom tailoring of platinum nanocatalysts for high-performance multifunctional electrocatalysis. *Nat. Catal.* **2**, 495-503 (2019).

25. Wang, Q. et al. Design of active nickel single-atom decorated MoS_2 as a pH-universal catalyst for hydrogen evolution reaction. *Nano Energy*. **53**, 458-467 (2018).

26. Zhang, J. et al. Engineering water dissociation sites in MoS_2 nanosheets for accelerated electrocatalytic hydrogen production. *Energy Environ. Sci*. **9**, 2789-2793 (2016).

27. Jiang, K. et al. Single platinum atoms embedded in nanoporous cobalt selenide as electrocatalyst for accelerating hydrogen evolution reaction. *Nat. commun*. **10**, 1743 (2019).

28. Zhang, H. et al. Implanting isolated Ru atoms into edge-rich carbon matrix for efficient electrocatalytic hydrogen evolution. *Adv. Energy Mater*. **10**, 2000882 (2020).
