# Supplementary material for: Tailoring coordination environments of single-atom electrocatalysts for hydrogen evolution by topological heteroatom transfer
Source: Nat Commun. 2024 Mar 30;15:2774. doi: 10.1038/s41467-024-47061-6 (PMC10981667; doi:10.1038/s41467-024-47061-6)
Supplement: Supplementary file 3 — Description of Additional Supplementary Files [file 41467_2024_47061_MOESM3_ESM.pdf]

Description of Additional Supplementary Files

Supplementary Movie 1: A corresponding movie of the Co–P_2N_2–C based AEM electrolyser operates at applied cell voltage of 1.7 V.
